# Mutational signature-based identification of DNA repair deficient gastroesophageal adenocarcinomas for therapeutic targeting

**DOI:** 10.1038/s41698-024-00561-6

**Published:** 2024-04-08

**Authors:** Aurel Prosz, Pranshu Sahgal, Brandon M. Huffman, Zsofia Sztupinszki, Clare X. Morris, David Chen, Judit Börcsök, Miklos Diossy, Viktoria Tisza, Sandor Spisak, Pornlada Likasitwatanakul, Orsolya Rusz, Istvan Csabai, Michael Cecchini, Yasmine Baca, Andrew Elliott, Peter Enzinger, Harshabad Singh, Jessalyn Ubellaker, Jean-Bernard Lazaro, James M. Cleary, Zoltan Szallasi, Nilay S. Sethi

**Affiliations:** 1 Danish Cancer Institute, Copenhagen, Denmark; 2 Department of Medical Oncology, Dana-Farber Cancer Institute, Boston, MA USA; 3 Department of Medicine, Brigham and Women’s Hospital and Harvard Medical School, Boston, MA USA; 4 Computational Health Informatics Program, Boston Children’s Hospital, Boston, MA USA; 5 Broad Institute of Massachusetts Institute of Technology (MIT) and Harvard University, Cambridge, MA USA; 6 Division of Gastrointestinal Oncology, Department of Medical Oncology, Dana-Farber Cancer Institute, Boston, MA USA; 7 Temerty Faculty of Medicine, University of Toronto, Toronto, ON Canada; 8 Institute of Molecular Life Sciences, HUN-REN Research Centre for Natural Sciences, Budapest, Hungary; 9 2nd Department of Pathology, SE NAP, Brain Metastasis Research Group, Semmelweis University, Budapest, Hungary; 10 Department of Physics of Complex Systems, Eötvös Loránd University, Budapest, Hungary; 11 Department of Medical Oncology, Center for Gastrointestinal Cancers, Yale Medical Center, New Haven, CT USA; 12 Caris Life Sciences, Phoenix, AZ USA; 13 Department of Molecular Metabolism, Harvard T.H. Chan School of Public Health, Boston, MA USA; 14 Department of Radiation Oncology, Dana-Farber Cancer Institute, Boston, MA USA; 15 Center for DNA Damage and Repair (CDDR), Dana-Farber Cancer Institute, Boston, MA USA; 16 Department of Bioinformatics and Department of Pathology, Forensic and Insurance Medicine, Semmelweis University, Budapest, Hungary

**Keywords:** Cancer genomics, High-throughput screening

## Abstract

Homologous recombination (HR) and nucleotide excision repair (NER) are the two most frequently disabled DNA repair pathways in cancer. HR-deficient breast, ovarian, pancreatic and prostate cancers respond well to platinum chemotherapy and PARP inhibitors. However, the frequency of HR deficiency in gastric and esophageal adenocarcinoma (GEA) still lacks diagnostic and functional validation. Using whole exome and genome sequencing data, we found that a significant subset of GEA, but very few colorectal adenocarcinomas, show evidence of HR deficiency by mutational signature analysis (HRD score). High HRD gastric cancer cell lines demonstrated functional HR deficiency by RAD51 foci assay and increased sensitivity to platinum chemotherapy and PARP inhibitors. Of clinical relevance, analysis of three different GEA patient cohorts demonstrated that platinum treated HR deficient cancers had better outcomes. A gastric cancer cell line with strong sensitivity to cisplatin showed HR proficiency but exhibited NER deficiency by two photoproduct repair assays. Single-cell RNA-sequencing revealed that, in addition to inducing apoptosis, cisplatin treatment triggered ferroptosis in a NER-deficient gastric cancer, validated by intracellular GSH assay. Overall, our study provides preclinical evidence that a subset of GEAs harbor genomic features of HR and NER deficiency and may therefore benefit from platinum chemotherapy and PARP inhibitors.

## Introduction

Platinum agents are essential for the chemotherapy treatment of gastrointestinal cancers^1^. There are several biological mechanisms that result in solid tumors being sensitive to platinum-based treatments including DNA repair pathway aberrations, such as homologous recombination (HR) deficiency or nucleotide excision repair (NER) deficiency. Platinum agents are highly mutagenic and generate both inter- and intrastrand DNA lesions. The HR repair pathway corrects DNA double-strand breaks created by lesions such as platinum-induced interstrand crosslinks. Alterations in HR genes are associated with increased platinum sensitivity in multiple tumor types including breast and ovarian cancer^2,3^. HR deficiency is likely to be present in gastric and esophageal adenocarcinoma (GEA) since mutations in key HR genes, albeit with low frequency, have been detected, for example, in gastric adenocarcinoma^4,5^.

In addition to driving sensitivity to platinum-based agents, HR deficiency forms a synthetic lethal relationship with small molecule inhibitors of poly (ADP ribose)-polymerase (PARP), and PARP inhibitors are now FDA approved for HR-deficient breast, ovarian, prostate, and pancreatic tumors. Initial clinical trials of PARP inhibitors in gastric cancer showed mixed results, with a second-line regimen of Olaparib/paclitaxel showing benefit compared to paclitaxel/placebo in a molecularly unselected population, as well as in ATM deficient patients, in a phase 2 but not in the confirmatory phase 3 GOLD trial^6,7^. Suboptimal patient selection may have contributed to the disappointing results seen in the phase 3 study, especially since BRCA2 deficiency was associated with significant Olaparib response in patient derived xenografts^8^. Patients on the GOLD trial had progressed on first-line chemotherapy, which typically includes platinum chemotherapy; whereas a key biomarker for PARP inhibitor sensitivity in ovarian and pancreatic cancer patients has been platinum sensitivity^9^. In addition, it is widely agreed that new genomic markers of PARP inhibitor sensitivity in GEA are needed, and a goal of ongoing clinical trials is to identify and molecularly define the subpopulation of GEA that are sensitive to PARP inhibition (NCT03008278).

HR deficiency is found in tumors without canonical HR gene mutations, and such cases are detectable by the presence of HR deficiency-associated mutational patterns (signatures). HR deficiency induces multiple types of genetic alterations ranging from single nucleotide variations to large scale genomic rearrangements. The presence and frequency of these events can be used to calculate clinically applicable mutational signatures, such as the HRD score^10^. High levels of these HR deficiency indicators are associated with better response to PARP inhibitor therapy in ovarian cancer^11^.

Here, we applied a validated genomic signature of HR deficiency, analogous to the FDA approved HRD score, to characterize the landscape of HR deficiency in gastrointestinal cancers. We found that a significant fraction of GEA has elevated levels of HR deficiency signatures and that the frequency of HR deficiency in GEA is higher than predicted based on *BRCA1/2* mutational status alone. We unexpectedly found a human GEA cell line model with outlier cisplatin sensitivity that exhibited NER deficiency. We then applied our recently published complex mutational signature of NER deficiency^12^ to next-generation sequencing data and found that NER deficient cases are likely present in GEA. To characterize cisplatin response in GEA NER deficiency, we performed single-cell transcriptional profiling on our human GEA NER deficient cell line model following chemotherapeutic stress, nominating ferroptosis as an additional mechanism of cancer cell death.

## Results

### Frequency of HR deficiency in GEA whole exome sequencing cohorts

To evaluate the frequency of HR deficiency in gastrointestinal cancers, we analyzed whole exome sequencing (WES) and whole genome sequencing (WGS) data from patient samples. WGS data contains about a 100-fold greater number of HR deficiency-induced mutational events than WES data^13^. In addition, certain genomic aberrations, such as large-scale rearrangements, can be detected with high confidence only by WGS. However, significantly more cases of WES data are available for analysis compared to WGS data. After removing MSI cases, there were 316 gastric cancer WES cases in TCGA, 68 gastric cancer WGS cases from PCAWG; there were 177 esophageal cancer WES cases in TCGA, 97 esophageal cancer WGS cases in PCAWG and 419 colorectal WES cases in TCGA versus 42 colorectal WGS cases in PCAWG (Supplementary Table 1). We therefore began our analysis using WES cohorts.

Mutations in the canonical HR genes *BRCA1*, *BRCA2* and *PALB2* are present in 3−5% of GEA tumors^14^; however, only a fraction of these cases have biallelic loss with both a predicted loss-of-function mutation and loss of heterozygosity (LOH) of the wild-type (WT) allele (Supplementary Figs. 1–4). Since LOH of the WT allele is typically required to confer HR deficiency^15^ in the setting of a *BRCA1/2* mutation, we considered only those *BRCA1/2* mutant cases that had biallelic mutations or a mutation accompanied by LOH to be HR deficient.

**Fig. 1:**
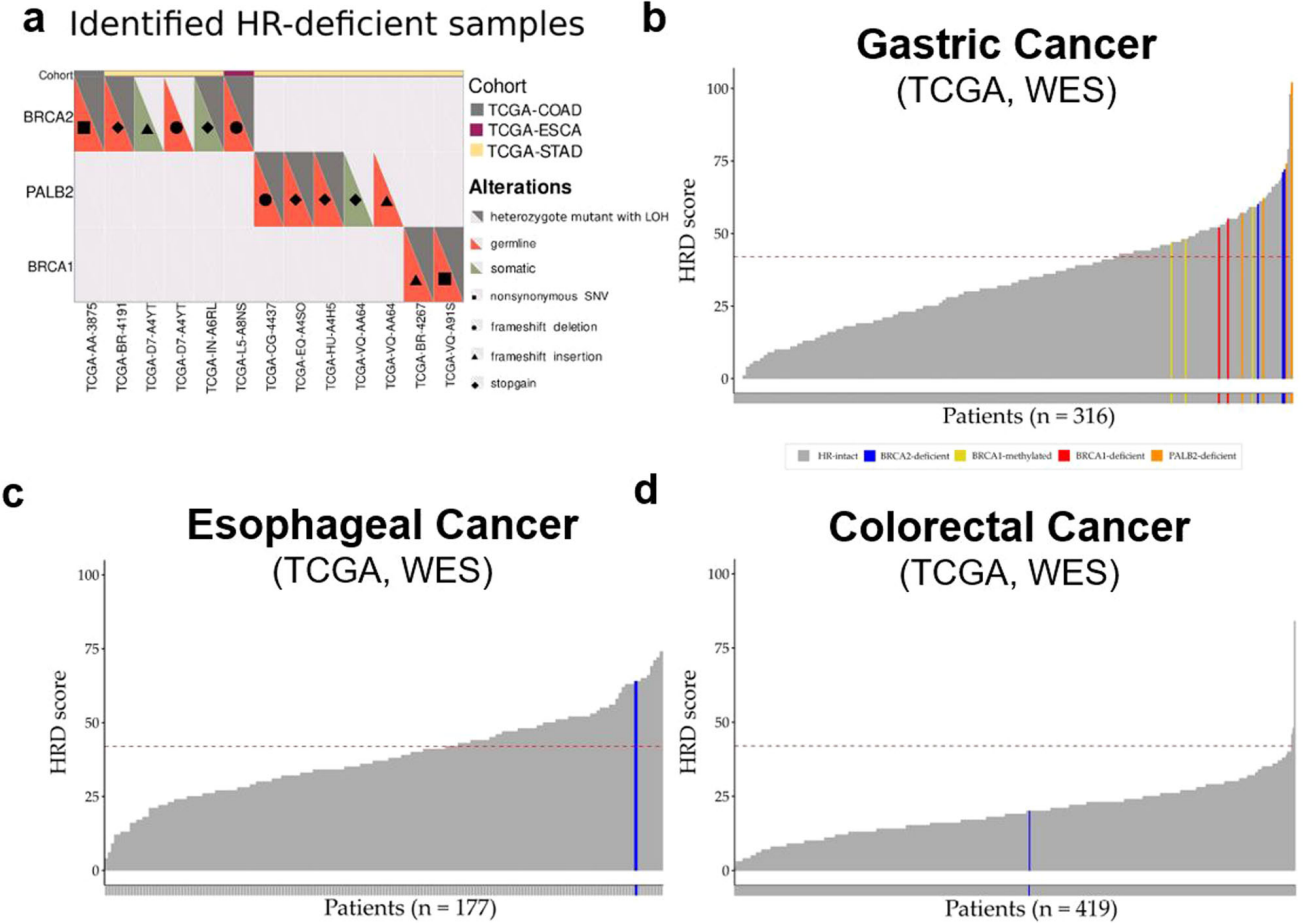
HRD mutational signature in gastrointestinal malignancies. **a** Germline and somatic mutations and copy number alterations of HR genes across the TCGA-STAD, TCGA-ESCA, TCGA-COAD and TCGA-READ WES cohorts. HRD score distribution in the TCGA-STAD (**b**), TCGA-ESCA (**c**), TCGA-COAD and TCGA-READ (**d**) WES samples. Cut-off value of ≥42 for HR deficiency was previously defined (dashed-line).

In the TCGA gastric cancer WES dataset, we identified two cases with a pathogenic *BRCA2* mutation accompanied by LOH, one *BRCA2* case where both alleles had pathogenic mutations, and two cases with pathogenic *BRCA1* mutation accompanied by LOH (Fig. 1, Supplementary Table 2). We also found four pathogenic *PALB2* mutations accompanied by either LOH or biallelic mutations in the TCGA gastric cancer cohort (Fig. 1, Supplementary Table 2). In the TCGA esophageal and colorectal WES data sets, we identified one case in each cohort with a pathogenic *BRCA2* mutation accompanied by LOH.

We next determined mutation and LOH status for DNA damage checkpoint genes such as *ATM* (1.2% in GEA), *ATR* (0.2% in GEA), *TP53* (28% in GEA), and *RB1* (0.8% in GEA) (Supplementary Figs. 1–4), although loss of function of these genes is usually not associated with an HR deficiency mutational signature^16^. *TP53* was the most frequently altered DNA damage checkpoint genes in the cohorts. We also identified three gastric adenocarcinoma cases with significant BRCA1 promoter methylation (Fig. 1). These analyses indicate that a small but significant fraction of gastrointestinal cancers harbor mutations in genes that function in DNA repair and DNA damage response.

### Genomic scar-based HRD scores in gastrointestinal cancer

The first FDA approved genomics-based method to quantify the degree of HRD was derived based on data obtained from hybridization microarrays. It has three components: (1) The HRD-LOH score is calculated by tallying the number of LOH regions exceeding 15 Mb in size but less than the whole chromosome^17^; (2) the Large-scale State Transitions (LST) score^18^ is defined as the number of chromosomal breaks between adjacent regions of at least 10 Mb, with a distance between them not larger than 3 Mb; and (3) the number of Telomeric Allelic Imbalances (TAI)^19^ is the number of AIs (unequal contribution of parental allele sequences) that extend to the telomeric end of a chromosome. These measures were later adapted to next generation sequencing and the sum of these scores is referred to as the HRD score^10^, which was recently approved by FDA as a companion diagnostic for prioritizing patients with ovarian cancer for PARP inhibitor therapy.

In the TCGA WES gastric cancer cohort, three *BRCA2* deficient, four *PALB2* deficient, two *BRCA1* deficient and three *BRCA1* promoter methylated cases all had an HRD score ≥42 (Fig. 1a, *p* < 1 × 10^-5^, Fisher exact test), which is the threshold for HR deficiency previously defined for ovarian cancer^10^. However, there were an additional 89 of 316 (28%) cases without a *BRCA1/2* or other HR gene mutation that also had an HRD score ≥42 (Fig. 1b*)*. Out of these 89 samples, only four cases could be explained by TOP2A mutation associated genomic instability^20^ (Supplementary Fig. 5). Notably, of the three molecular subtypes of gastric adenocarcinoma included in our analysis (MSI cases were excluded)^4,5^, substantially more cases with an HRD score ≥42 belonged to the subgroup with chromosomal instability, in contrast to the genomically stable and EBV positive subtypes (Supplementary Fig. 6).

In the TCGA WES esophageal cancer cohort, a *BRCA2* deficient case demonstrated an HRD score ≥42 (Fig. 1c). Similar to the TCGA gastric cancer cohort, there were an additional 68 of 179 cases (40%) without a *BRCA1/2* or other HR gene mutation that also had an HRD score larger than the threshold. Given the high frequency of HR deficiency in GEA, we analyzed the prevalence of HR deficiency in another common gastrointestinal malignancy, colorectal cancer. However, unlike GEA, HR deficiency was rare in colorectal cancer. In a cohort of 419 colorectal cancer cases, only 3 had an HRD score ≥42, suggesting that HRD is rare in this cancer type (Fig. 1d).

Determination of HRD scores from WES data can be impacted by the relatively low number of SNVs (24) ; therefore, we also calculated HRD scores from WGS data for the 32 cases that had both WGS and WES data available in the TCGA STAD cohort. Similar to other solid tumors, the WES and WGS derived HRD scores showed a strong correlation (*R* = 0.87; *p* = 1.4 × 10^-10^, Supplementary Fig. 7).

Since only a minority of cases with high levels of HR deficiency associated mutational signatures harbored a loss of function in *BRCA1/2* or other HR genes, we investigated whether the detected HR deficiency could be explained by other mechanisms. Suppression of HR gene function can occur via other mechanisms such as promoter methylation of HR genes, such as *BRCA1* in breast cancer, or regulatory proteins such as *RBBP8*^21–23^. However, in our analyses, we were unable to find evidence of promoter methylation that could explain the increased HRD scores, and other HR deficiency associated mutational signatures (Supplementary Fig. 8). We found no other HR related genes whose promoter methylation was associated with increased HRD scores in the analyzed cohorts.

### High HRD score by mutation signature analysis correlates with platinum and PARP inhibitor sensitivity in gastric cancer cell lines

**Fig. 2:**
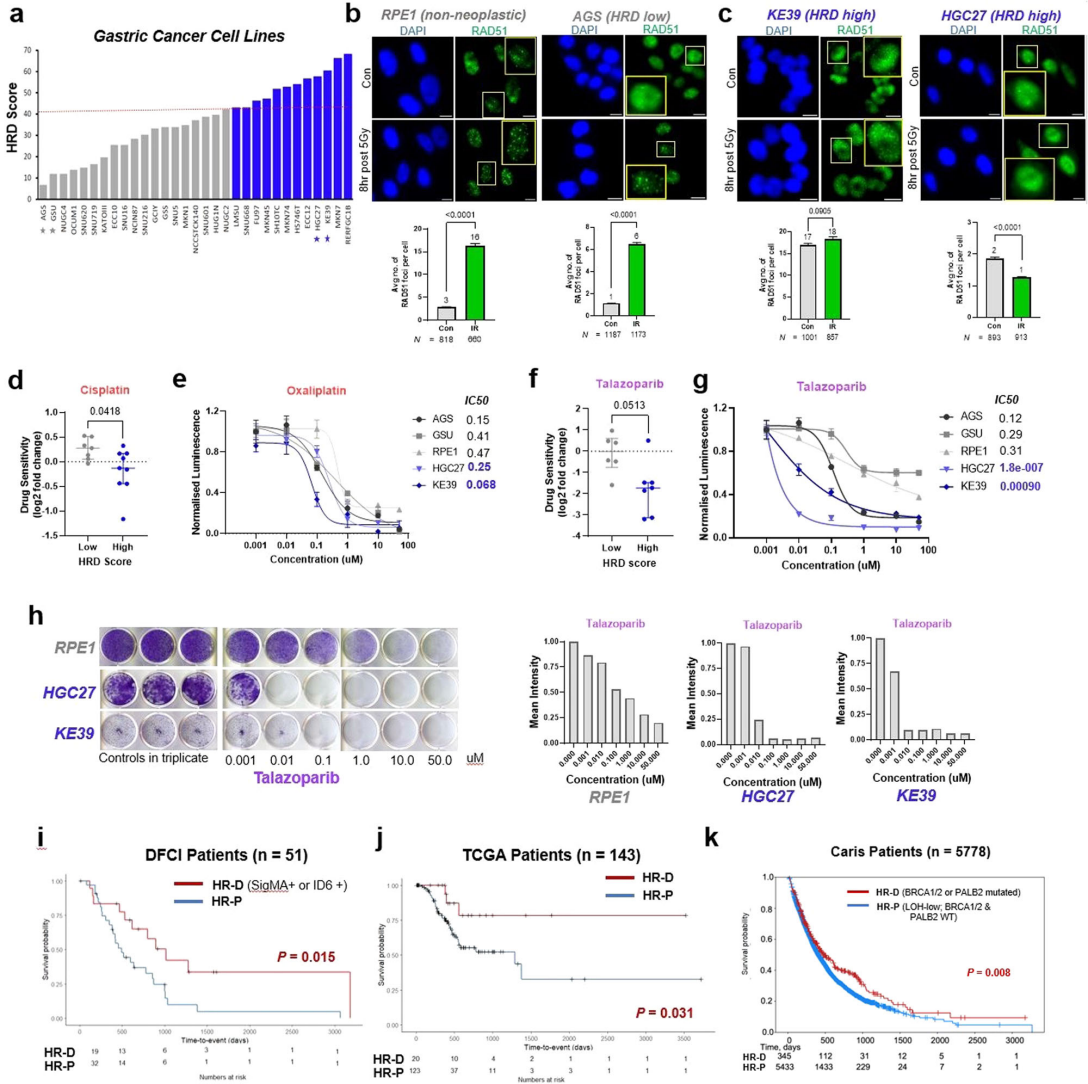
High HRD scores by mutation signature analysis is associated with platinum and PARP inhibitor sensitivity in gastric cancer. **a** Gastric cancer patients cell lines from CCLE stratified by HRD Score. Cut-off value of ≥42 for HR deficiency was previously defined (dashed-line); cell-lines with low (gray) and high (blue) HRD scores indicated. Starred cell-lines used in experimental validation studies. **b** Representative immunofluorescence images (top) and quantification (bottom) for RPE1 and AGS cells; Number of RAD51 (green) positive foci indicating DNA repair after treatment with 5 Gy gamma radiation; Data expressed as the mean number of RAD51 positive foci per cell ±SEM; Scale bar = 10 µm. **c** Representative immunofluorescence images (top) and quantification (bottom) from KE39 and HGC27 cell lines; Number of RAD51 (green) positive foci indicating DNA repair after treatment with 5 Gy gamma radiation; Data expressed as the mean number of RAD51 positive foci per cell ±SEM; Scale bar = 10 µm. **d** Cisplatin sensitivity between low (gray) and high (blue) HRD gastric cancer cell lines available in BROAD institute PRISM repurposing drug screen dataset. Difference between the cisplatin sensitivity (log2 fold change) is represented as the median with interquartile range. **e** Dose-response curve of non-neoplastic RPE1, low (gray) and high (blue) HRD (blue) gastric cancer cell lines to oxaliplatin. Error bars indicate SD. Best-fit IC50 scores are displayed. **f** Talazoparib sensitivity between low (gray) and high (blue) HRD gastric cancer cell lines available in BROAD institute PRISM repurposing drug screen dataset. Difference between the cisplatin sensitivity (log2 fold change) is represented as the median with interquartile range. **g** Dose-response curve of non-neoplastic cell line RPE1, low (gray) and high (blue) HRD gastric cancer cell lines to indicated concentrations of Talazoparib. Error bars indicate SD. Best-fit IC50 scores are displayed. **h** Colony formation assay (left) and the quantification shown as mean intensity from each well using ImageJ (right) of gastric cancer cell with high HRD score (blue) and non-neoplastic RPE1 (gray). **i** Platinum-specific survival in patients treated at Dana-Farber Cancer Institute with metastatic gastroesophageal adenocarcinoma who have Sigma 3 signature >0 or Cosmic ID6 signature >0 versus Sigma 3 signature = 0 and Cosmic ID6 signature = 0 (median platinum-specific survival 1015 days versus 496 days, HR = 2.43 [95% CI: 1.16−5.08], log-rank *p* = 0.0152). X axis, time from first dose of platinum-based chemotherapy in days; Y axis, proportion surviving. **j** Platinum-specific survival in patients from the TCGA-STAD cohort with localized gastric adenocarcinoma, who have HRD score >=42 and HRDetect score >= 0.7 (HR-D group) versus HRD score < 42 and HRDetect score <0.7 (HR-P group). Median platinum-specific survival 1015 days versus 496 days, HR = 3.39 [95% CI: 1.05−11.01], log-rank *p* = 0.0308. X axis, time from first dose of platinum-based chemotherapy in days; Y axis, proportion surviving, progression-free survival. **k** Overall survival in a multicenter cohort of patients with microsatellite stable gastroesophageal cancer who have a pathogenic/likely pathogenic mutation in BRCA1, BRCA2, or PALB2 versus those with Loss of Heterozygosity-Low (LOH-low) score (score < 16%) (median overall survival: 459 days versus 354 days, HR = 0.809 [95% CI: 0.677−0.968], log-rank *p* = 0.02). X axis, time from diagnosis in days; Y axis, proportion surviving.

To evaluate whether putative HR deficient gastric cancer cases, as identified by mutational signatures, are in fact HR deficient, we calculated the HRD score for 31 gastric cancer cell lines and stratified them by increasing HRD score. An HRD score cutoff of 42 was used to divide cell lines into high and low categories for further investigation (Fig. 2a). It is worth noting, similar to patient data (Fig. 1), high HRD score cells lines do not necessarily harbor alterations in BRCA1/2 or other categories of HR-related genes (Supplementary Fig. 9). Next, to test whether gastric cancer cell lines with high HRD scores are functionally HR deficient, we employed a RAD51 foci functional assay, which assesses HR proficiency by interrogating the ability of RAD51 foci to form in response to double strand DNA (dsDNA) breaks. We chose two high HRD (KE39 and HGC27) and two low HRD (AGS and GSU) gastric cancer cell lines to perform the assay (Fig. 2a). In addition to these gastric cell lines, we utilized RPE1 as a non-neoplastic, HR-proficient cell line control. RPE1 cells and low HRD cell lines induced RAD51 foci in response to the gamma radiation-induced DNA damage, indicating functional HR (Fig. 2b). Both high HRD cell lines did not display gamma radiation-induced RAD51 foci formation, confirming HR deficiency (Fig. 2c and Supplementary Fig. 10A). To confirm these results using an alternative method of DNA damage induction, we used radiomimetic DNA-cleaving agent phleomycin and evaluated the change in phospho-H2AX (pH2AX) and RAD51 expression, which are surrogates for dsDNA breaks and HR activity, respectively. RPE1 and GSU did not display DNA damage (change in pH2AX expression) or changes in RAD51 levels, suggesting either faster repair or insufficient DNA damage, precluding the assessment of HR proficiency. AGS, KE39 and HGC27 displayed robust pH2AX activity in response to phleomycin treatment. While AGS displayed induction of RAD51 expression, KE39 and HGC27 did not, indicating HR deficiency (Supplement Fig. 10B, C). Collectively, these results suggest that high HRD status indicated by mutation signature analysis can identify gastric cancer cell lines with functional HR pathway deficiency.

Our and others’ prior work have shown that HR-deficient ovarian, breast, and prostate cancers are exquisitely sensitive to platinum agents and PARP inhibition^11^^,^^13,22–24^, and we hypothesized that gastric cancers deficient in HR (or with high HRD scores) would be more sensitive to platinum agents and PARP inhibition. To test this hypothesis, we first used the chemotherapeutic agents’ sensitivity data from the Broad Institute’s PRISM repurposing drug screen^25^. Gastric cancer cell lines were divided into two groups/quartiles based on low and high HRD scores (Fig. 2a). The sensitivity to most chemotherapeutic agents is not significantly different between these two groups (Supplementary Fig. 11). Consistently, gastric cancer cell lines evaluated in this study did not show differential sensitivity to 5-FU treatment (Supplementary Fig. 12A). Although most chemotherapy agents did not display a differential impact on viability, one exception to this was that platinum chemotherapy preferentially reduced viability of high HRD gastric cancer lines (Fig. 2d), which we confirmed for HGC27 cells treated with cisplatin (Supplementary Fig. 12B). Closer examination of oxaliplatin treated cell lines showed a subset of high HRD ones with greater sensitivity (Supplementary Fig. 11, red box). Consistently, luminescence-based viability assays indicated that HR-deficient KE39 and HGC27 were predominantly more sensitive to oxaliplatin (Fig. 2e). These results suggest that HR-deficient gastric cancer display greater sensitivity to platinum agents.

We next investigated whether HR-deficient gastric cancer cell lines are sensitive to PARP inhibition. Humans have at least three important PARP family members (PARP1/2/3), and each PARP inhibitor has different potency, with talazoparib (BMN-673) exhibiting the most potency^26–28^. Using Broad PRISM drug sensitivity data, we found that high HRD lines showed greater sensitivity to many PARP inhibitors compared to low HRD ones (Supplementary Fig. 13), with talazoparib showing the most potent effect (Fig. 2f). We confirmed that high HRD lines showed exquisite sensitivity to talazoparib compared to low HRD ones (Fig. 2g). Olaparib, rucaparib, AZD2461, and veliparib also showed greater activity in high HRD lines (Supplementary Fig. 12C−F). Since the duration of PARP inhibition is important^29^, we also performed two-week colony formation assays with talazoparib and veliparib, showing at least a 100-fold greater sensitivity in high HRD lines compared to RPE-1 (Fig. 2h and Supplementary Fig. 12G).

### HRD mutational signatures are associated with response to platinum treatment in patients with GEA

We next examined whether patients with HR deficient gastric cancers as determined by inactivating mutations of HR genes and/or the HRD-associated mutational signature had clinical benefit from platinum chemotherapy. First, we analyzed a cohort of patients with GEA (Supplementary Table 3) who were treated with platinum-based chemotherapy at Dana-Farber Cancer Institute (DFCI) and whose tumors were profiled by Oncopanel, a targeted sequencing panel covering 447 cancer-associated genes. HRD status was determined using SigMA^30^, which uses SNVs derived by Oncopanel as input, and also by detecting ID6^31^, a short deletion signature associated with HR deficiency. Consistent with our experimental results, platinum-treated GEA patients with HR deficiency displayed significantly better overall survival (Fig. 2i). Next, we analyzed the TCGA cohort of patients with GEA who received platinum treatment. Patients with gastric cancers exhibiting an HRD score >42 displayed significantly better overall survival compared to patients with HRD low tumors (Fig. 2j). Finally, we analyzed a large cohort of GEA patients whose tumors were genomically profiled by Caris Inc. For this cohort, we only had access to a limited amount of genomic data, namely, mutation status of key HR genes (BRCA1, BRCA2, PALB2) and the genomic LOH score as determined by FoundationFocus CDxBRCA, an FDA-approved diagnostic to detect HR deficiency. We classified patients as HR deficient if they had evidence of biallelic inactivation with loss-of-function mutations and LOH in BRCA1, BRCA2, or PALB2; HR proficient if they had no such mutations and had a low FoundationFocus CDxBRCA genomic LOH score as specified by the manufacturer. Consistent with the first two patient cohorts, Caris-profiled patients with HR deficient GEA had a significantly better overall survival. Thus, all three cohorts showed a consistent clinical picture in that patients with HR deficient GEA benefitted from treatment with platinum chemotherapy.

### NER deficient gastric cancer cell line displays cisplatin and PARP inhibitor sensitivity

**Fig. 3:**
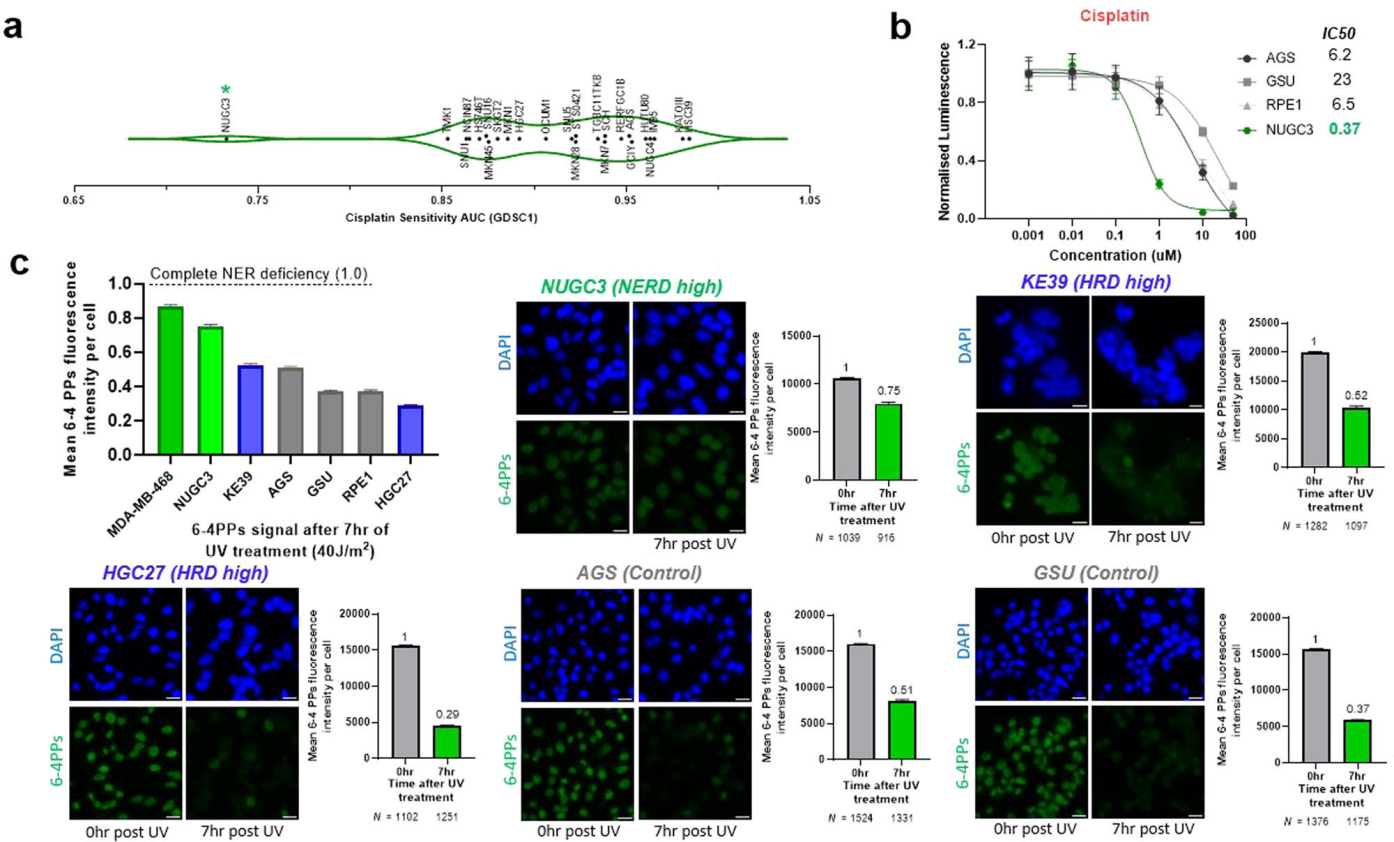
Cisplatin sensitivity analysis reveals NER deficient gastric cancer cell line. **a** Cisplatin sensitivity in gastric cancer cell lines from Sanger’s Genomics of Drug Sensitivity in Cancer Project (GDSC1); drug response measured as Area Under the Curve (AUC); NUGC3 (green star). **b** Cisplatin dose-response curve of NUGC3 (green) and three control cell lines (gray). Best-fit IC50 scores are displayed. **c** Gastric cancer cell lines showing varying degrees of nucleotide excision repair deficiency by the functional assay monitoring the cells’ ability to remove 6-4-photoproducts. NER activity is expressed by the amount of 6-4-photoproducts removed post 7 h 40 J/m^2^ of UV irradiation. Complete NER deficiency is indicated as 1. Representative immunofluorescence images (left) and quantification (right) of 6-4 photoproducts (6-4PPs- green), monitoring the cells’ ability to remove 6-4-photoproducts and assessing NER. NER activity is expressed by the loss of 6-4PPs signal post 7 h of 40 J/m^2^ UV irradiation. Data expressed as mean ± SEM; Scale bar = 20 µm.

Based on the experimental confirmation that HR-deficient gastric cancers are sensitive to platinum chemotherapy, we wondered whether HR-deficient cell lines could be identified by sensitivity to cisplatin. To this end, we analyzed Sanger’s Genomics of Drug Sensitivity in Cancer Project (GDSC)^32^, searching for gastric cancer cell lines with high cisplatin sensitivity. Among gastric cancer cell lines, NUGC3 displayed the highest sensitivity to cisplatin by a significant margin (Fig. 3a). Luminescent-based viability assay and colony formation assay confirmed strong cisplatin sensitivity of NUGC3 (Fig. 3b and Supplementary Fig. 14A). Compared to cisplatin, oxaliplatin and 5-fluorouracil (5-FU) had less impact on NUGC3 proliferation (Supplementary Fig. 14B−D), consistent with GDSC data (Supplementary Fig. 14E).

We next evaluated whether NUGC3 is functionally HR deficient by the RAD51 foci assay. Upon 5 Gy gamma radiation exposure, NUGC3 displayed an increase in RAD51 foci formation (Supplementary Fig. 14F). Moreover, phleomycin treatment yielded robust induction of pH2AX that corresponded to RAD51 induction (Supplementary Fig. 14G). Together, these results suggest that NUGC3 is HR proficient.

We therefore asked whether another DNA repair pathway was defective in NUGC3 cells to explain the outlier sensitivity to cisplatin. NER is a critical DNA repair pathway involved in the repair of UV radiation-induced DNA damage and cisplatin-induced DNA damage^33,34^. Inactivation of this pathway, through deleterious *ERCC2* mutations, is associated with cisplatin sensitivity in bladder cancer^35^. To investigate whether NUGC3 cells are NER deficient, we first employed the 6-4 pyrimidine-pyrimidone photoproducts (6-4PPs) assay, which monitors the ability to remove UV-induced 6-4PPs by the cell. 6-4PPs can be removed by both global genome repair (GGR) and transcription coupled repair (TCR) NER pathways and their removal is closely correlated with NER efficiency^36,37^. NUGC3 and MDA-MB-468, an NER-deficient breast cancer cell line^33^, displayed a significantly reduced capacity to remove 6-4PP photoproducts 7 h post UV irradiation (Fig. 3c), indicating NER deficiency, whereas all other cell lines tested (RPE1, GSU, AGS, HGC27, and KE39) were adept at removing 6-4PP and therefore deemed NER proficient (Fig. 3c and Supplementary Fig. 14H). To confirm these results, we performed a second proteo-probe assay involving damaged-DNA binding protein 2 (DDB2), an essential initiation factor with preferential activity in GGR NER pathway^38^ that binds to UV-induced 6,4-photoproduct. Similar to 6-4PP assay, the DDB2 assay measures repair of 6,4-PPs. Unlike other cell lines tested, NUGC3 cells did not fully repair 6,4-photoproducts as indicated by retained DDB2 signal 150 min post UV exposure (Supplementary Fig. 14I), suggesting a functional deficiency in NER. Collectively, these data indicate that NUGC3 is functionally NER deficient and that GEA tumors can be NER deficient.

Since NUGC3 cells were unable to completely repair UV-induced 6,4-photoproducts, we next asked whether NUGC3 cells are selectively sensitive to UV radiation. Compared to RPE1 cells, NUGC3 cells displayed a dose-dependent sensitivity to UV radiation exposure by colony formation assay (Supplementary Fig. 15A). These findings suggests that a subset of gastric cancers are NER deficient and display heightened sensitivity to cisplatin-containing regimens.

To provide a potential molecular explanation for NER deficiency in NUGC3 cells, we analyzed the mutational profiles of these cell lines. Consistent with HR proficiency in NUGC3 cells, we did not observe mutations in common HR genes like *BRCA1/2, PALB2, ATM, FANCA*. By contrast, we observed nonsynonymous mutations in genes that function in GGR and TCR NER pathways^34^ (Supplementary Fig. 15B). Of all surveyed gastric cancer cell lines in the CCLE, NUGC3 is the only gastric cancer cell line harboring mutations in essential NER pathway genes (Supplementary Fig. 15C). NUGC3 cells also showed the lowest expression of *XPC*, a gene that plays an essential role during the first step of GG-NER (Supplementary Fig. 15D). Interestingly, lower expression of XPC correlates with better cisplatin sensitivity in gastric, ovarian, colorectal, and lung cancer^39–43^. It is worth mentioning that the only other NER-deficient cancer cell line (MDA-MB-468) reported previously, does not harbor mutations in NER genes; instead, NER deficiency in this cell line is driven by epigenetic silencing of *ERCC4*^33^. These results indicate that somatic mutations in essential NER pathway genes can contribute to functional NER deficiency in GEA.

PARP is not only a vital component of HR but also NER^44^. Several studies suggest efficacy of PARP inhibitors for ovarian and lung cancers harboring mutations in NER pathway genes (*ERCC1, ERCC8, DDB1, XAB2*)^45–47^. We, therefore, examined whether NER-deficient gastric cancer is sensitive to PARP inhibition using NUGC3 as our model. While most PARP inhibitors showed only modest activity, talazoparib showed potent, selective killing in NUGC3 cells compared to three control cell lines (Supplementary Fig. 16). These data suggest there may be a role for potent PARP inhibitors, such as talazoparib, in NER-deficient GEA.

### Identification of NER deficient gastrointestinal cancer cases in the clinical setting

**Fig. 4:**
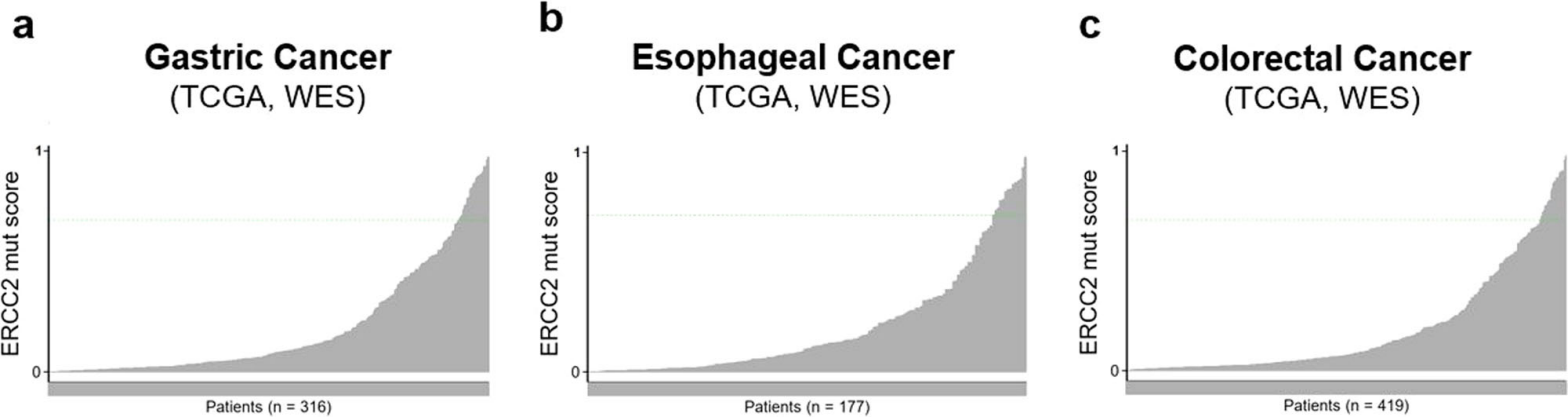
NER deficiency associated mutational signature score in gastrointestinal malignancies. The NER deficiency associated complex mutational signature was calculated in the WES data for gastric (**a**), esophageal (**b**) and colorectal (**c**) cancer WES data. The cut-off value was set to ≥0.7 for NER deficiency.

Despite the potential clinical actionability of tumor NER deficiency, there are currently no functional or IHC assays available to reliably identify NER deficiency in clinical specimens. Next-generation sequencing (NGS)-based mutational signatures can be used to identify NER deficiency. We recently identified a complex mutational signature, predominantly driven by the mutational signature SBS5 and ID8, that is significantly increased in NER deficient bladder cancer cases, especially those with *ERCC2* mutations^12^. We determined the values of the same complex mutational signature for the TCGA GEA cancer cases. When we used the same threshold value (>0.7) that distinguished NER deficient and proficient cases in bladder cancer, we found 22 gastric cancer cases, 14 esophageal cancer cases and 25 CRC cases with a > 0.7 value (Fig. 4). However, none of these cases had an inactivating mutation in the canonical NER genes (such as *ERCC2* and *ERCC3*). To determine whether this complex mutational signature may in fact reflect NER deficiency we turned to the genomic analysis of eight gastric adenocarcinoma organoids derived from patients at DFCI from the Cancer Cell Line Factory (CCLF). One of the tumors had inactivating mutations in *ERCC2* and *ERCC6*, two key genes of NER. The NER associated mutational signatures were the highest in this case, relative to other cases without inactivating mutations in NER genes (Supplementary Fig. 17). This was a 73-year-old man who had a locally advanced, poorly differentiated, microsatellite stable gastric adenocarcinoma. He was treated with 2 months of 5-FU, leucovorin and oxaliplatin (FOLFOX) and then underwent a total gastrectomy with D2 lymph node dissection. Pathology showed minimal response to neoadjuvant FOLFOX and the pathologic stage was ypT4N0. He then switched to docetaxel, cisplatin and 5-FU for a total of 4 cycles of adjuvant therapy. He is still alive today with no evidence of disease at the last imaging follow up, which was 4.5 years after completion of adjuvant chemotherapy. Consistent with our cell line data, this tumor was resistant to oxaliplatin-containing therapy, and perhaps the switch to cisplatin (in addition to the standard-of-care tumor resection) contributed to his excellent outcome. These data suggest patients with gastrointestinal cancer may have tumors with NER deficiency that can be detected by mutation signature analysis. Interestingly, for the same organoid case the calculated HRD score components were the lowest in the analyzed samples.

### scRNA-sequencing reveals ferroptosis as a cisplatin-specific mechanism of cell death in NER deficient gastric cancer

**Fig. 5:**
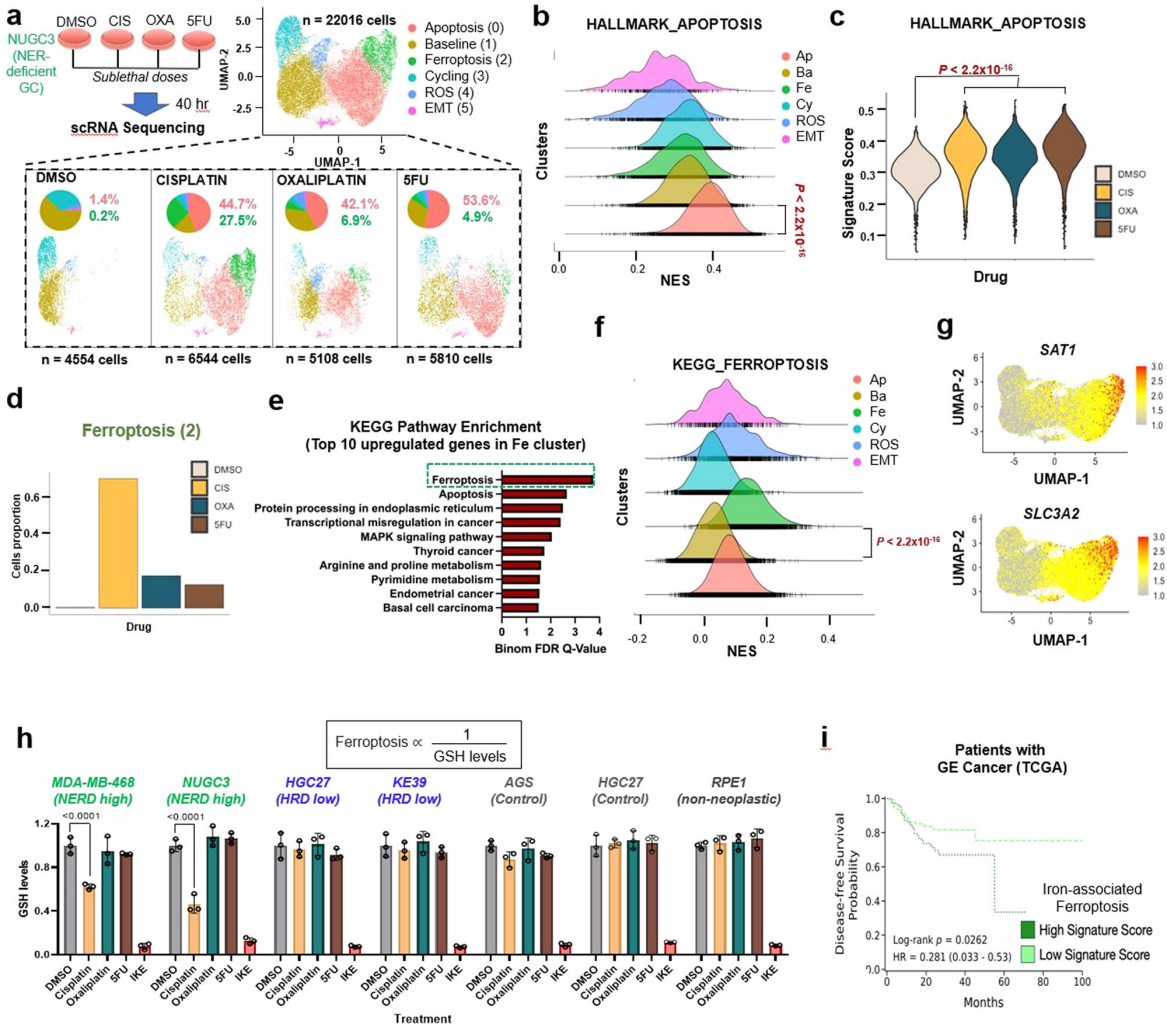
scRNA-seq analysis of NER deficient gastric cancer cells reveal distinguishing features of distinct chemotherapy treatments. **a** Experimental setup and UMAP representation of single cell transcriptome profiling of NUGC3 cells treated with DMSO (control), cisplatin, oxaliplatin or 5-FU colored by distinct cell clusters. UMAP of separated samples along with pie chart indicating distribution of cell clusters (bottom panel). **b** Single cell-gene set enrichment analysis (SC-GSEA) between apoptosis (0) cluster and rest of the clusters showing enrichment of HALLMARK_APOPTOSIS Pathway; NES: Normalized Enrichment Score. **c** Violin plots indicating expression of HALLMARK_APOPTOSIS pathway in the four treatment groups (EnrichR) of top 20 genes upregulated in cluster Apoptosis (0) (also Supplementary table 3). *P* value calculated by Wilcoxon rank sum test with Bonferroni correction. **d** Normalized cell counts in each treatment group for Ferroptosis (2) cluster. **e** Hallmark pathways enrichment analysis (EnrichR) of top 20 genes upregulated in Ferroptosis (2) cluster. **f** Single cell-Gene set enrichment analysis (SC-GSEA) between Ferroptosis (2) cluster and rest of the clusters showing enrichment of KEGG_FERROPTOSIS Pathway; NES: Normalized Enrichment Score. **g** UMAP representation of SAT1 (top) and SLC3A2 (bottom) gene expression. **h** Measurement of intracellular GSH levels as the proxy for ferroptosis in indicated cell lines after 40 h treatment of DMSO (control) or sublethal doses of cisplatin, oxaliplatin, 5-FU in addition to IKE (inducer of ferroptosis). Breast cancer cell line MDA-MB-468 is NER deficient (positive control). RPE1 is non-neoplastic cell line (negative control). All data expressed as mean ± SD luminescence signal normalized with the DMSO treatment for each cell line. **i** Kaplan Meier curve indicating disease-free survival of patients with GE cancer (TCGA, *n* = 593) whose tumors demonstrated high and low iron-associated ferroptosis signature.

To better understand the mechanism of increased sensitivity of NER-deficient gastric cancer to cisplatin, we performed single-cell RNA sequencing (scRNA-seq) on NUGC3 cells treated with sublethal doses of three different chemotherapeutics commonly used to treat gastric cancer: cisplatin, oxaliplatin or 5-FU (Fig. 5a and Supplementary Fig. 18A). UMAP representation of single cell gene expression profiles from 22,016 cells demonstrated six distinct clusters categorized based on gene ontology (Fig. 5a, Supplementary Fig. 19, Supplementary Tables 4 and 5). Cluster 0 (red) was induced in all three chemotherapy treatment groups (1.4% in DMSO; 44.7%, 42.1%, and 53.6% in cisplatin, oxaliplatin, and 5-FU treated, respectively); it showed a gene expression profile most significantly associated with apoptosis (Fig. 5a−c, and Supplementary Fig. 18B), which is consistent with a general effect of chemotherapy irrespective of NER. Based on transcriptional profiles, it appears that apoptosis might have been induced by TNFα/NF-κB and p53 signaling (Supplementary Fig. 18C−E). In contrast, cluster 2 (green) was preferentially induced in cisplatin-treated NUGC3 cells relative to the other chemotherapeutic agents (Fig. 5d; 27.5% in cisplatin compared to 6.9% in oxaliplatin and 4.9% in 5-FU). Pathway enrichment analysis indicated that ferroptosis was the top pathway enriched in cluster 2 (Fig. 5e, f). Indeed, *SAT1* and *SLC3A2*, two well-described ferroptosis genes^48–50^, were preferentially induced in cluster 2 (Fig. 5g and Supplementary Fig. 18F).

We next examined the status of TCR- and GG-NER genes, expecting that most would be silenced by way of genomic alteration (Supplementary Fig. 15C). Interestingly, expression of POLR gene family members, a principal component of the TCR-NER pathway, was upregulated in cluster 2 (green), with *POLR2L* showing the highest expression (Supplementary Fig. 18H, I). These results suggest that since GG-NER is compromised due to *XPC* deficiency, TCR-NER pathway components may be upregulated in a compensatory role attempting to facilitate NER. However, apart from POLR2L, which is unaltered and presumed to be functional in NUGC3 cells, the genes in the shared-NER pathway (*POLE* and *RPA1)* and downstream of TCR-NER pathway (*ERCC6* and *GTF2H1*) remain inactivated leading to functional NER deficiency (Supplementary Figs. 15B and 18G), especially in response to cisplatin (cluster 2).

To validate ferroptosis as an additional mechanism of cell death in NER-deficient cell line NUGC3 specifically after cisplatin treatment, we performed the intracellular GSH assay. Recent reports demonstrate that GSH depletion triggers ferroptosis^51,52^ and therefore can be used as a negatively correlated surrogate of ferroptosis. Imidazole ketone erastin (IKE) induces ferroptosis by reducing GSH levels and was used as a treatment positive control^53^. NUGC3, MDA-MB-468 (positive control cell for NER deficiency), RPE1(non-neoplastic), AGS/GSU (HR/NER proficient) and HGC27/KE39 (HR deficient) were treated with DMSO or sublethal doses of cisplatin, oxaliplatin and 5FU followed by the GSH assay. IKE reduced GSH in all the cell lines and triggered ferroptosis, indicating assay fidelity. Cisplatin treatment reduced GSH only in the NER-deficient cell lines- MDA-MB-468 and NUGC3, suggesting a selective induction of ferroptosis. Other agents (oxaliplatin and 5-FU) did not change GSH in any cell line (Fig. 5h). These results suggest that while general apoptosis is induced by all three chemotherapeutic agents, cisplatin treatment induces an additional mechanism of cell death through ferroptosis, potentially explaining the enhanced sensitivity of NUGC3 cells to cisplatin.

From our unbiased analyses of key ferroptosis-related genes (please see methods), we observed that the differentially expressed regulators and markers were enriched for those associated with iron metabolism, many of which exert protective effects (e.g., *ZFP36*)^54^. To establish a clinical association between ferroptosis and GE cancer, we evaluated the expression of iron-associated ferroptosis regulators as assessed by bulk RNA-sequencing data from TCGA esophageal and gastric cancer tumor samples and patient outcomes (*n* = 593). We compared the disease-free survival in patients with GE cancers whose tumors had high and low iron-associated ferroptosis expression signature. Here, we observed that a high iron-associated ferroptosis signature score was associated with poor disease-free survival outcome compared to a low score (Fig. 5i; HR = 0.281 (0.033−0.53), log-rank test *P* = 0.0262), suggesting that iron-associated ferroptotic mechanisms of cell death is in part associated with improved disease-free survival outcome in patients with GE cancer. In addition, we found that patient tumor samples with poorly differentiated Stage IV esophageal and gastric cancer showed a trend towards increased iron-associated ferroptosis signature score compared to Stage I cancer, although this was not a significant difference (one-tailed Mann Whitney Wilcoxon Test, *P* = 0.06). Finally, when breaking down the iron-associated ferroptosis gene signature into individual components, we found that expression of ferroptosis protector *ZFP36*^54^ alone portended worse patient outcomes (HR = 1.75 (1.3−2.36); *P* < 0.001).

## Discussion

HR deficiency is often driven by loss of BRCA1, BRCA2 or PALB2 function in tumor cells, which is the result of inactivating mutations and LOH. Consequently, many HR deficient cases are identified by sequencing *BRCA1/2* or *PALB2*, and this approach has led to initial approval of PARP inhibitors in multiple tumor types including breast, ovarian, pancreatic, and prostate cancer. There is clear evidence that functional HR deficiency can be achieved without mutations in known HR genes, principally *BRCA1/2* or *PALB2*. These mechanisms include loss or mutation of other HR genes (such as *RAD51C*) and suppression of *BRCA1* or other HR genes expression by methylation.

Mutational signature-based approaches have recently been applied to improve prediction of HR deficiency because they detect the consequences of HR deficiency rather than the underlying cause. The first diagnostic HR deficiency mutational signature (HRD score) was recently approved to direct PARP inhibitor therapy in ovarian cancer cases without *BRCA1/2* mutations based on data showing that patients with high HR deficiency associated mutational signatures without canonical HR gene mutations (*BRCA1*, *BRCA2*, etc.) benefited from PARP inhibitor therapy^11^. These findings raise the possibility that other tumor types with similar mutational signatures in the absence of core HRD mutations may also be functionally HR deficient and could benefit from PARP inhibitor therapy.

GEA is only partially characterized in terms of DNA repair pathway aberrations. We find that *BRCA1/2* loss-of-function mutations – when present in both alleles or when coupled with LOH – are associated with the same HR deficiency associated mutational signatures as are present in *BRCA1/2*-mutant ovarian, breast, or prostate tumors. Therefore, *BRCA1/2* mutant GEA, although relatively rare, appear to have bona fide HR deficiency.

In addition to the small percentage of HR-deficient GEA cases due to loss of *BRCA1/2*, we also found that a significant number of GEAs with WT *BRCA1/2* showed levels of HRD associated mutational signatures as high as those observed in *BRCA1/2* deficient cases. Reliable detection of HR-deficient GEA may have important clinical implications. Patients with HR-deficient tumors may be prioritized for platinum-based chemotherapy^11,55^.

HR deficiency associated mutational signatures were first identified in breast and ovarian cancer, which harbor the highest frequency of *BRCA1/2* inactivating events. We now show that the same signatures may also be useful in identifying HR deficiency in GEA, a tumor type with far less frequent alterations in *BRCA1/2*. These findings may have implications for PARP inhibitor clinical trials and suggest that mutational analysis of known HR genes such as *BRCA1/2* could be combined with mutational signature approaches (such as the FDA approved HRD score) to identify cases most likely to harbor functional HR deficiency. Among PARP inhibitors tested, talazoparib consistently showed better sensitivity compared to first-generation PARP inhibitors^29,56^. Additional studies will be required to define mechanisms of HR deficiency in GEA, optimize threshold values of the HR deficiency mutational signatures for clinical application, and understand the therapeutic implications of HR deficiency in GEA.

We also found that NER deficiency is present in GEA. An NER deficient cell line NUGC3 has loss of function mutations in the genes that participate in GGR (*XPC, CHD1L, MCR51*) and TCR NER pathways. While XPC is necessary for the first repair step of GG-NER, RNA polymerase II encoded by *POLR2L* is necessary for the first repair step of TCR-NER^57^. NUGC3 cells have loss of function mutation in *XPC*, but *POLR2L* is wild type though other downstream genes of *POLR2L* involved in subsequent TCR-NER repair steps have loss of function mutations, e.g., *ERCC6* and *GTF2H1*^58^. The shared-NER pathway also carries loss of function mutations (*POLE and RPA1*). Interestingly, The *CHD1L* mutation found in NUGC3 cell line is a pathogenic (score 0.91) breast cancer somatic mutation (Genomic Mutation ID COSV63615250). We have observed exceptional GEA tumor responses to platinum chemotherapy and wonder whether NER deficiency underlie these cases.

There is increasing interest in understanding the relationship between ferroptosis and cancer, both from a vulnerability and drug resistance standpoint^59^. Recent studies have shown that cisplatin induces ferroptosis by depleting GSH levels in lung cancer and colorectal cancer cell lines (A549 and HCT116) and synergizes with ferroptosis inducer erastin (IKE) for better anti-cancer response^60,61^. We found that ferroptosis is an additional mechanism of cell death associated with cisplatin but not 5-FU or oxaliplatin chemotherapy in NER-deficient GEA. Indeed, the anti-tumor effects of oxaliplatin and cisplatin have differed, leading to preferential treatment of distinct cancers; cisplatin being used for breast and lung cancers while oxaliplatin incorporated into gastrointestinal cancer treatment regimens. A study showed that oxaliplatin’s anti-tumor activity depends on ribosome biogenesis stress whereas cisplatin relies on DNA-damage^62^. Our results support cisplatin’s distinct anti-tumor properties and nominate selective activation of ferroptosis as a potential reason. Further validation across a broader array of GEA models will support this observation.

Our study has some limitations. (1) As opposed to WGS, the limitations of using WES samples are fewer available variants for the mutational signature extraction part, decreasing the reliability of the extracted features. (2) The extracted mutational signatures are limited to those previously described within TCGA-STAD, TCGA-ESCA, TCGA-COAD, and TCGA-READ cohorts, which raises the possibility of missing novel signatures. (3) The extracted mutational signatures and genomic scar scores only inform about the historical state of the tumors, and do not engender information about possible acquired drug resistance mechanisms. (4) We used six gastric cancer cell lines to validate HR/NER deficiency and drug sensitivity. The validation can be scaled up to a high-throughput fashion using automated imaging, incorporating more GEA cell lines in future studies. Furthermore, futures studies should incorporate newer models, such as organoids. (5) We did not combine chemotherapeutic agents and PARP inhibitors in our studies; combinations may show better sensitivity than monotherapy but may manifest unnecessary toxicity. (6) Since NER deficiency appears to be rare, we only had one cell line model to study. Additional models when recognized should be examined to validate our findings. (7) Lastly, our patient data analysis would benefit from better clinical annotation (e.g., therapy duration); confirmation that patients with GEA that harbor deficiency in HR and NER pathways are more responsive to platinum-based chemotherapy should follow. Furthermore, clinical trials involving GEA and PARP inhibitors should be evaluated for relationship between HR/NER deficiency and tumor response (NCT03008278, NCT01123876).

## Methods

### Genotyping

Germline and somatic variants in WGS samples were downloaded from the ICGC data portal (https://dcc.icgc.org/). Whole exome somatic and germline vcf files were downloaded from the TCGA data portal (https://portal.gdc.cancer.gov/). The high fidelity of the reported germline and somatic variants was ensured by the application of the tools default filters (FILTER == “PASS”). The high fidelity of the reported germline and somatic variants was ensured by the application of additional hard filters on the somatic samples: TLOD ≥ 6, NLOD ≥ 3, NORMAL.DEPTH ≥ 15, TUMOR.DEPTH ≥ 20, TUMOR.ALT ≥ 5, NORMAL.ALT = 0, TUMOR.AF ≥ 0.05. The pathogenicity of the variants was assessed by Intervar (version 2.0.2) which classifies variants into five categories: “Benign”, “Likely Benign”, “Uncertain significance”, “Likely Pathogenic” and “Pathogenic”. Mutations in exonic regions that were not synonymous SNVs and classified as “Pathogenic” or “Likely Pathogenic” were considered as deleterious^12^.

In order to estimate tumor cellularity and ploidy and to infer allele-specific copy number (ASCN) profiles Sequenza^63^ was used. The fitted models were in the ploidy range of [1, 7] and cellularity range of [0, 1]. When the predictions of a fitted model were significantly different from the 2 expected ploidy and cellularity values, an alternative solution was selected manually. If the copy numbers of either the A or B alleles dropped to zero within the coordinates of a gene, then an LOH event was registered. The final genotypes were determined as: Wild type: no pathogenic or likely pathogenic germline or somatic mutation(s). Wild type with LOH: no pathogenic or likely pathogenic germline or somatic mutation(s), but an LOH event occurred. Heterozygote mutant: a pathogenic or likely pathogenic germline or somatic mutation is present, but no LOH. Heterozygote mutant with LOH: a pathogenic or likely pathogenic germline or somatic mutation is present and an LOH event occurred. Homozygote mutant: an identical germline or somatic mutation is present in both alleles. Compound heterozygote mutant: two different germline and/or somatic mutations are present in both alleles^12^.

The genotyping of the samples was performed as shown (Supplementary Figs. 1–4). The samples with identified *BRCA1/2* and other HR related pathogenic gene mutations are listed in Supplementary Table 1.

### Mutational signature extraction

Single base substitution (SBS) signatures were extracted with the help of the deconstructSigs R package (https://cran.r-project.org/web/packages/deconstructSigs/index.html) which determines the linear combination of pre-defined signatures that most accurately reconstructs the mutational profile of a single tumor sample. The selected signatures, the linear combination of which could lead to the final mutational catalog, were confined to those, that were reported to be present in both gastric, esophageal and colorectal cancer according to the Catalog of Somatic Mutations in Cancer (COSMIC) (https://cancer.sanger.ac.uk/cosmic/signatures_v2), and signature 3 were also extracted along with them. After evaluation of a sample’s signature composition, its mutational catalog was reconstructed, and the cosine of the angles between the 96-dimensional original and reconstructed vectors was calculated (cosine similarity)^12,13^.

Structural variants (SVs) for germline and somatic samples were downloaded from the ICGC data portal. The resulting structural variants in each sample were mapped to a 32-dimensional rearrangement signature (RS) catalog described in breast cancer (M). The previously identified matrix of rearrangement signatures (P) was downloaded from the following link: https://static-content.springer.com/esm/art%3A10.1038%2Fnature17676/MediaObjects/41586_2016_BFnature17676_MOESM47_ESM.zip. As previously, the M and the P matrices were used in a non-negative least-squares problem to estimate the matrix of exposures to mutational processes^12,13^.

Doublet base substitution and indel signatures were extracted with the ICAMS R package (https://cran.rstudio.com/web/packages/ICAMS/index.html)^12,13^.

For the gastric cell line samples, the variants were obtained from the DepMap portal (https://depmap.org/portal/download/), and the mutational signature and genomic scar score analysis was performed the same way as the patient samples. For the genotyping, the non-conserving mutations were excluded, and only the damaging were kept.

### Genomic scar scores

The three genomic scar scores were calculated for each sample with the help of the scarHRD R package^64^ from the copy number profile of the samples, and the sum of these scores were used as the final HRD score for a sample.

### Ferroptosis gene expression signature analysis

Previous work has established a representative, curated list of ferroptosis-related genes^65^. We used this list in tandem with FerrDb^66^ to categorize the genes as pro- or anti-ferroptotic to perform an unbiased analysis of ferroptosis-related regulators and markers. From our unbiased analyses, it was apparent that many of the differentially regulated regulators and markers were associated with iron metabolism. To determine the association of the iron-associated ferroptosis regulators and markers with tumor grade, we calculated the gene set enrichment analysis (GSEA) score of the gene expression signature for the iron-associated ferroptosis targets, as defined by FerrDb. Specifically, we analyzed the gene expression of ACO1, CD44, CISD1, IREB2, NCOA4, NFS1, PHKG2, STEAP3, TFRC, ZFP36, and ZEB1 in the normalized RNA-sequencing data from TCGA esophageal and gastric cancer tumor samples with tumor grade annotations (*n* = 593). TCGA RNA-sequencing data of tumor samples was normalized to RNA-sequencing data of paired normal samples and z-scored for sample-wise normalization. Statistical outliers of the iron-associated signature scores were removed using Tukey’s fence method, where outliers were considered larger than 1.5 times the interquartile range above the third quartile or below 1.5 times the interquartile range below the first quartile.

First, we stratified the remaining quality-filtered samples (*n* = 588) based on tumor grade and compared the grade-specific iron-associated ferroptosis signature scores of poorly differentiated tumor samples (Stage II, II, IV) to well differentiated tumor samples (Stage I) using a one-tailed Mann-Whitney Wilcoxon Test to detect a positive shift in iron-associated ferroptosis signature score distribution. Second, we identified the subset of patients with disease-free survival outcomes (*n* = 327) and compared the disease-free survival outcome of the top 40% (*n* = 131) and the bottom 40% (*n* = 131) tumor samples stratified based on iron-associated ferroptosis signature score. Log-rank test (α < 0.05) was used to test for differences in the probability of disease events between iron-associated ferroptosis signature high and iron-associated ferroptosis signature low populations. Cox proportional hazard ratio was used to estimate the effect size of the association between the iron-associated ferroptosis signature and disease-free survival outcome.

### Cell lines and cell culture

Human gastric cancer cell lines were obtained from the Cancer Cell Line Encyclopedia (CCLE) core facility (BROAD Institute, Cambridge), which obtained them directly from commercial sources and authenticated the lines using standard short tandem repeat analysis. RPE and MDA-MB-468 cells were obtained from ATCC. RPE1 cells were grown in DMEM-F12 (Life Technologies, #10565042) supplemented with 10% FBS and 1% penicillin/streptomycin, HGC27 cells were grown in DMEM (Life Technologies, #11965118) supplemented with 10% FBS and 1% penicillin/streptomycin. AGS, KE39, GSU, NUGC3 and MDA-MB-468 were grown in RPMI 1640 (Life Technologies, #11875119) supplemented with 10% FBS and 1% penicillin/streptomycin. Cells lines were maintained in humidified 37 °C the incubator with 5% CO2 and routinely tested for mycoplasma contamination (Lonza #LT07-118).

### Cell proliferation assay

For IC50 experiments, 1000 cells were plated in a flat-bottom 96-well plate. Cells were treated with either vehicle (DMSO) or different concentrations of chemotherapeutic agents or PARP inhibitors. Luminescence was measured using CellTiter-Glo (Promega, #G7572) for ATP amount after 3-5 days, and final readings were normalized with Day 1 luminescence readings. For colony formation assay, 2 × 10^4−1 × 10^5 cells were plated in 6-well plates. Cells were then treated with DMSO or inhibitors, and treatments were renewed every 3-4 days (different doses of UV radiation for UV damage assay using StrataLinker 2400 irradiator). After 7−10 days, cells were fixed in 1% paraformaldehyde for 15 min at RT, washed twice with PBS, and stained with 0.1% crystal violet solution in ethanol (Sigma Aldrich, #HT901) for 15 min at room temperature. After washing the plate with distilled water and drying them, the plates were scanned with a document scanner and the confluency of cells per well was quantified using ImageJ. Briefly, the RGB .tif images were converted to 8-bit greyscale images. ‘oval’ selection in imageJ was used to select each well precisely and ‘measure’ command was used to measure the mean signal intensity from each well. Intensity value from an empty well (background) was subtracted from each well. Finally, the control wells’ intensity was used to normalize the acquired signal values for drug treated wells and plotted as graphs.

### In silico drug sensitivity analysis

To evaluate drug sensitivity data for gastric cancer cell lines, PRISM drug repurposing data were downloaded 22Q2 data release from DepMap website of Broad Institute (https://depmap.org). The cell lines were divided into a high or low group based on quartiles of HRD score. The difference between the drug sensitivity for the two groups was plotted as median with interquartile range, and the *p*-value was calculated using the Mann Whitney test.

### Single-cell RNA sequencing sample preparation

NUGC3 cells were treated with the IC50 concentration of Cisplatin, Oxaliplatin, 5-FU and DMSO for 2 days. The cells were washed with PBS, trypsinized and labeled with cell hashing antibodies, TotalSeq^TM^-C0251 Hashtag 1 (BioLegend #394661) and TotalSeq^TM^-C0252 Hashtag 2 (BioLegend #394662). Viable cells were washed and resuspended in PBS with 0.04% BSA at a cell concentration of 1000 cells/µL. About 17,000 viable mouse cells were loaded onto a 10× Genomics Chromium^TM^ instrument (10× Genomics) according to the manufacturer’s recommendations. The scRNAseq libraries were processed using Chromium^TM^ single cell 5’ library & gel bead kit (10× Genomics). Matched cell hashing libraries were prepared using single cell 5’ feature barcode library kit. Quality controls for amplified cDNA libraries, cell hashing libraries, and final sequencing libraries were performed using Bioanalyzer High Sensitivity DNA Kit (Agilent). The sequencing libraries for scRNAseq and scTCRseq were normalized to 4 nM concentration and pooled using a volume ratio of 4:1. The pooled sequencing libraries were sequenced on Illumina NovaSeq S4 300 cycle platform. The sequencing parameters were: Read 1 of 150 bp, Read 2 of 150 bp and Index 1 of 8 bp. The sequencing data were demultiplexed and aligned to mm10-3.0.0 using cell ranger version 3.1.0 pipeline (10× Genomics).

### Single-cell RNA sequencing

#### General analysis

scRNA-seq IntegrateData function in Seuratv4 was used to counteract batch effects among human samples. Principal Component Analysis (PCA) was then completed on the integrated object and the quantity of principal components selected for clustering was determined using the integrated object’s elbow plot. Cells were then visualized primarily using UMAP non-linear dimensional reduction from which feature and violin plots were generated to demonstrate distribution of gene expression and expression levels of various marker genes and gene signatures throughout the population.

#### Pre-processing, alignment and gene counts

De-multiplexing, alignment to the transcriptome, and unique molecular identifier (UMI) collapsing were performed using the Cellranger toolkit provided by 10X Genomics.

#### General clustering

Standard procedures for QC filtering, data scaling and normalization, detection of highly variable genes, and hashtag oligo (HTO) demultiplexing were followed using Seurat v4 in RStudio. Cells with unique feature counts lower than 500 and greater than 7500 as well as cells with greater than 15% mitochondrial DNA were excluded. Counts were log-normalized and scaled by a factor of 10,000 according to the default parameters when using the Seurat LogNormalize function. Variable features were identified, and the data were scaled using the default parameters (Ngenes = 2000) of the FindVariableFeatures Extended Data Fig. ScaleData Seurat functions, respectively. HTOs were demultiplexed using the HTODemux function, and cells were identified as containing HTO-1 or HTO-2 based on their maximal HTO-ID signal. The cell population was filtered to contain only HTO-positive, singlet cells for further analysis. Principle component analysis (PCA) was completed on the remaining cells and 10 principle components were selected for clustering, tSNE, and UMAP analyses. Cells were visualized primarily using UMAP non-linear dimensional reduction (dims 1:10, resolution = 0.3), from which feature plots were generated to demonstrate distribution of gene expression and different drugs treatment cells and expression levels of various marker genes throughout the population. Marker genes for each resulting cluster were found using the FindMarkers function with the minimum prevalence set to 25%. Cluster identities were defined using known marker genes enriched in different pathways.

#### scRNA-seq gene signature analysis

To analyze existing gene signatures on our scRNA-seq data, the Seurat AddModuleScore function in Seurat v4 was used to calculate the average normalized and scaled gene expression of a given gene list in each individual cell. Specific cell types were identified using established marker genes and gene signatures. Gene signature scoring was then visualized with feature and violin plots. To generate novel gene signatures, the Seurat FindMarkers function was used to create lists of genes differentially expressed in one specified subset in comparison to another given subset. Minimum prevalence was set to 25%.

### GSH assay

Intracellular GSH levels were measured using luminescence-based GSH-Glo™ Glutathione Assay kit (Promega #V6911). In this assay, luciferin derivative is converted into luciferin in the presence of GSH in a reaction that is catalyzed by glutathione S-transferase. The amount of light generated in the firefly luciferase-coupled reaction is proportional to the amount of GSH present in the sample. The assay was performed according to the manufacturer’s instructions. Briefly, 1000 cells were plated in flat-bottom 96-well plates and the following day, treated with the drugs. After approximately 40 h, the media was removed and GSH-Glo™ reagent was added in each well, followed by 30 min incubation. 100 μL reconstituted luciferin detection reagent was added and incubated for another 15 min. The bioluminescence was measured with the plate reader. Imidazole ketone erastin IKE (Selleckchem #S8877) was used as the positive control for ferroptosis. The level of ferroptosis is inversely proportional to the levels of GSH.

### Patients and cohorts

In this study, 791 whole genome and whole exome sequenced pretreatment samples were analyzed from four cohorts of patients with GEA. We analyzed 68 cases of gastric cancer and 97 cases of esophageal cancer with WGS data and 441 cases of gastric cancer and 185 cases of esophageal cancer with WES data (Supplementary Table 1). For the analysis, the normal, tumor bam and vcf files were downloaded from the ICGC data portal (https://dcc.icgc.org/) for the WGS, and from the TCGA data portal (https://portal.gdc.cancer.gov/) for the WES samples. The WES vcf files were generated by MuTect2 (GATK, v3.8).

Next generation sequencing (DFCI Oncopanel) was analyzed from 102 cases of GEA using vcf files. Platinum-based survival was calculated by time from starting platinum chemotherapy to last follow up (*n* = 19 patients) or death (*n* = 68 patients) before filtering. Kaplan-Meier estimates were calculated for molecularly defined patient cohorts with significance determined as *p* < 0.05 by log-rank analysis.

Next-generation sequencing (592-gene or whole exome) was performed for GEA patient samples (*N* = 5863) submitted to Caris Life Sciences (Phoenix, AZ). Patients were stratified by the presence or absence of pathogenic/likely pathogenic (P/LP) mutations in the NER-related gene, ERCC2, and HR-related genes (BRCA1, BRCA2, PALB2). High genomic loss-of-heterozygosity (LOH-High) was defined as LOH at ³ 16% of examined loci. Real-world overall survival was obtained from insurance claims data and calculated as the time from biopsy to last follow up or death. There were 40% of patients who were censored in the HR-D cohort and 39% of patients who were censored in the HR-P cohort. Kaplan-Meier estimates were calculated for molecularly defined patient cohorts with significance determined as *p* < 0.05 by log-rank analysis.

### DNA methylation analysis

DNA methylation data measured by the Illumina HumanMethylation450 platform were downloaded from the TCGA data portal (https://portal.gdc.cancer.gov/)^67^. The median beta values of promoter-associated probes in a given gene were used.

### DDB2 proteo-probe assay for NER Deficiency

The DDB2 proteo-probe assay was performed based on publications^33,68^. Briefly, the purified HA-tagged DDB2 protein complex was used as a probe in an affinity fluorescence-based assay. Cells were plated on glass-teflon microscope slide (Tekdon, #518plain) and the next day exposed to 20 J/m2 UV-C at 254 nm using a StrataLinker 2400^TM^ irradiator. 5 or 150 min following UV exposure, cells were fixed with ice-cold methanol for 10 min at room temperature. After serial rehydration with phosphate-buffered saline (PBS), non-specific binding sites were blocked by incubation with PBS-0.3% BSA. DDB2 proteo-probe was diluted in PBS-BSA and added to the fixed cells for 2 h at 37 °C. Cells were then washed twice with PBS, and rabbit anti-HA antibody (1:600; CST #3724 S) was used to label the hybridized DDB2 proteo-probe. Cells were then rewashed with PBS, and goat anti-rabbit antibody coupled to Alexa fluor488 fluorochrome (1:600; Lifetech #A11008) was added. Following two final washes in PBS and one in purified water, coverslips were mounted in Fluoro-Gel II containing DAPI (EMS #17985-51).

### 6-4PPs assay for NER deficiency

Removal of 6-4 pyrimidine-pyrimidone photoproducts (6-4PPs) as a functional readout of NER was quantified using the immunofluorescent assay and is based on^69^. Briefly, the cells were grown on chamber slides (Ibidi #80826) overnight before irradiating with 40 J/m2 UV using StrataLinker 2400. Post irradiation, the cells were fixed with cold methanol for 10 min on the ice at 0 min and 7 h time points. Fixed cells were permeabilized with 0.5% triton X in PBS for 4 min at RT and incubated at 37 °C for 15 min in 2 M HCL in PBS. After washing twice with PBS, once with 1% BSA/PBS, and once with PBS, cells were incubated with 6-4PP primary antibody (Cosmo Bio #NM-DND-002, 1:2000) for 45 min at 37 °C followed by incubation with fluorescent secondary antibody and DAPI for 30 min at 37 °C. The slides were washed twice with PBS before imaging with Nikon Eclipse Ti2 Series inverted microscope (×40 objective). ImageJ was used to quantify the 6-4PPs signal from each cell.

#### RAD51 foci assay for HR deficiency

Cells were plated on glass-teflon microscope slide (Tekdon, #518plain) and the next day treated with either DMSO or Phleomycin (InvivoGen # ant-ph-1) for 1 h. For gamma irradiation, the cells were either kept in the cell culture hood or treating with 5 Gy IR radiation (10−15 min). Cells were then washed and fixed with 4% paraformaldehyde (PFA) for 15 min at RT, blocked and permeabilized PBS-BSA + 0.3% Triton X-100 for 15 min at RT, and incubated with primary antibodies (Rad51 Rabbit 1:200, CST # 8875 and pH2A.X Mouse 1:600 CST #80312) in PBS-BSA overnight at +4 °C. After three washes with PBS, secondary antibodies; anti-Rabbit IgG Alexa Fluor 488 (Life tech #A11008) and anti-Mouse IgG Alexa Fluor 568 (Life tech #A11004), diluted 1:300 in PBS-BSA with DAPI (1:1000) for 2 h at RT. Finally, the cells were washed with PBS three times, mounted, and covered in aluminum foil for imaging. Immunofluorescence imaging was performed using Nikon Eclipse Ti2 Series inverted microscope (×40 objective). NIS-Elements AR software was used to acquire the images. DAPI channel was used to focus the cells, and the images for 488 (RAD51) and 567 (pH2AX) channels were acquired, keeping the same exposure across the samples. The RAD51 positive foci were counted using the ‘find maxima’ and ‘analyze particles’ function of imageJ. To measure the mean fluorescence intensity of RAD51 and pH2AX from each cell, the 16-bit images were converted to 2-bit images in ImageJ, followed by ‘hole filling’ and segmentation (watershed) commands. The cells were then automatically recognized and counted using ‘analyze particles’, and the file was saved as an ‘image mask’. Finally, the image mask was overlaid on the original 488 (RAD51) and 567 (pH2AX) split channels, and the ‘measure’ command was used to get the mean fluorescence intensity from each cell.

### Ethics statement

This study was conducted in accordance with guidelines set forth in the Declaration of Helsinki, Belmont report, and U.S. Common rule. Per 45 CFR 46.104(d)(4)(ii) (July 19, 2018) for retrospective studies using deidentified biospecimens and clinical data, this study was considered IRB exempt and informed consent was not required. The Dana-Farber cohort study was approved under Dana-Farber Harvard Cancer Center IRB protocol #03-189 which all included participants signed informed consent to participate.

### Reporting summary

Further information on research design is available in the Nature Research Reporting Summary linked to this article.

### Supplementary information

Reporting Summary

Supplementary Material

Supplementary Table 4

Supplementary Table 5

## Data Availability

All the sequencing data and reagents (e.g., cell lines, inhibitors, antibodies) will be shared either by depositing in public domains or through MTA agreements in compliance with our institutions. The sequencing data generated by this manuscript have been deposited in GEO (GSE256301). The datasets analyzed during the current study are available in the National Cancer Institute GDC data portal (https://portal.gdc.cancer.gov/), the DepMap data portal (https://depmap.org/portal/), the Cancer Cell Line Factory (https://cellfactory.broadinstitute.org/), and the TCGA data portal (https://portal.gdc.cancer.gov/). Results shown here are based in part from data generated by the TCGA Research Network: http://cancergenome.nih.gov/ and the International Cancer Genome Consortium (ICGC): https://icgc.org/. The results presented in the current publication are based in part on the use of study data downloaded from the dbGaP web site.

## Code availability

There are no restrictions to access the custom code used for the analyses presented in this study. Information is available from the authors on request.
