# Author Correction: Mutational signature-based identification of DNA repair deficient gastroesophageal adenocarcinomas for therapeutic targeting

**DOI:** 10.1038/s41698-024-00587-w

**Published:** 2024-05-06

**Authors:** Aurel Prosz, Pranshu Sahgal, Brandon M. Huffman, Zsofia Sztupinszki, Clare X. Morris, David Chen, Judit Börcsök, Miklos Diossy, Viktoria Tisza, Sandor Spisak, Pornlada Likasitwatanakul, Orsolya Rusz, Istvan Csabai, Michael Cecchini, Yasmine Baca, Andrew Elliott, Peter Enzinger, Harshabad Singh, Jessalyn Ubellaker, Jean-Bernard Lazaro, James M. Cleary, Zoltan Szallasi, Nilay S. Sethi

**Affiliations:** 1 Danish Cancer Institute, Copenhagen, Denmark; 2 Department of Medical Oncology, Dana-Farber Cancer Institute, Boston, MA USA; 3 Department of Medicine, Brigham and Women’s Hospital and Harvard Medical School, Boston, MA USA; 4 Computational Health Informatics Program, Boston Children’s Hospital, Boston, MA USA; 5 Broad Institute of Massachusetts Institute of Technology (MIT) and Harvard University, Cambridge, MA USA; 6 Division of Gastrointestinal Oncology, Department of Medical Oncology, Dana-Farber Cancer Institute, Boston, MA USA; 7 Temerty Faculty of Medicine, University of Toronto, Toronto, ON Canada; 8 Institute of Molecular Life Sciences, HUN-REN Research Centre for Natural Sciences, Budapest, Hungary; 9 2nd Department of Pathology, SE NAP, Brain Metastasis Research Group, Semmelweis University, Budapest, Hungary; 10 Department of Physics of Complex Systems, Eötvös Loránd University, Budapest, Hungary; 11 Department of Medical Oncology, Center for Gastrointestinal Cancers, Yale Medical Center, New Haven, CT USA; 12 Caris Life Sciences, Phoenix, AZ USA; 13 Department of Molecular Metabolism, Harvard T.H. Chan School of Public Health, Boston, MA USA; 14 Department of Radiation Oncology, Dana-Farber Cancer Institute, Boston, MA USA; 15 Center for DNA Damage and Repair (CDDR), Dana-Farber Cancer Institute, Boston, MA USA; 16 Department of Bioinformatics and Department of Pathology, Forensic and Insurance Medicine, Semmelweis University, Budapest, Hungary

**Keywords:** Cancer genomics, High-throughput screening

Correction to: *npj Precision Oncology* 10.1038/s41698-024-00561-6, published online 08 April 2024

In this article, the author name Andrew Elliott was incorrectly written as Andrew Elliot. The original article has been corrected.

