# Supplementary material for: Mutational signature-based identification of DNA repair deficient gastroesophageal adenocarcinomas for therapeutic targeting
Source: NPJ Precis Oncol. 2024 Apr 8;8:87. doi: 10.1038/s41698-024-00561-6 (PMC11001913; doi:10.1038/s41698-024-00561-6)
Supplement: Supplementary file 1 — Reporting Summary [file 41698_2024_561_MOESM1_ESM.pdf]

nature portfolio

Corresponding author(s): Zoltan Szallasi, Nilay S. Sethi

Last updated by author(s): Feb 6, 2024

Reporting Summary

Nature Portfolio wishes to improve the reproducibility of the work that we publish. This form provides structure for consistency and transparency in reporting. For further information on Nature Portfolio policies, see our Editorial Policies and the Editorial Policy Checklist.

Statistics

For all statistical analyses, confirm that the following items are present in the figure legend, table legend, main text, or Methods section.

n/a	Confirmed	
☐	☒	The exact sample size (*n*) for each experimental group/condition, given as a discrete number and unit of measurement
☒	☐	A statement on whether measurements were taken from distinct samples or whether the same sample was measured repeatedly
☐	☒	The statistical test(s) used AND whether they are one- or two-sided<br>*Only common tests should be described solely by name; describe more complex techniques in the Methods section.*
☐	☒	A description of all covariates tested
☐	☒	A description of any assumptions or corrections, such as tests of normality and adjustment for multiple comparisons
☐	☒	A full description of the statistical parameters including central tendency (e.g. means) or other basic estimates (e.g. regression coefficient) AND variation (e.g. standard deviation) or associated estimates of uncertainty (e.g. confidence intervals)
☐	☒	For null hypothesis testing, the test statistic (e.g. *F*, *t*, *r*) with confidence intervals, effect sizes, degrees of freedom and *P* value noted<br>*Give P values as exact values whenever suitable.*
☒	☐	For Bayesian analysis, information on the choice of priors and Markov chain Monte Carlo settings
☒	☐	For hierarchical and complex designs, identification of the appropriate level for tests and full reporting of outcomes
☐	☒	Estimates of effect sizes (e.g. Cohen's *d*, Pearson's *r*), indicating how they were calculated

Our web collection on statistics for biologists contains articles on many of the points above.

Software and code

Policy information about availability of computer code

Data collection	No specific software was used to collect data. Please see methods on "Cell lines and cell culture", "Cell Proliferation Assay", "Single cell RNA sequencing sample preparation", "Patients and cohorts".
Data analysis	Sequenza, Intervar (version 2.0.2), deconstructSigs, ICAMS, scarHRD , Seuratv4. There are no restrictions to access the custom code used for the analyses presented in this study. Information is available from the authors on request.

For manuscripts utilizing custom algorithms or software that are central to the research but not yet described in published literature, software must be made available to editors and reviewers. We strongly encourage code deposition in a community repository (e.g. GitHub). See the Nature Portfolio guidelines for submitting code & software for further information.

Data

Policy information about availability of data

All manuscripts must include a data availability statement. This statement should provide the following information, where applicable:

- Accession codes, unique identifiers, or web links for publicly available datasets
- A description of any restrictions on data availability
- For clinical datasets or third party data, please ensure that the statement adheres to our policy

All the sequencing data and reagents (e.g. cell lines, inhibitors, antibodies) will be shared either by depositing in public domains or through MTA agreements in compliance with our institutions. The datasets generated and/or analysed during the current study are available in the National Cancer Institute GDC data portal

(https://portal.gdc.cancer.gov/), the DepMap data portal (https://depmap.org/portal/), the Cancer Cell Line Factory (https://cellfactory.broadinstitute.org/), and the TCGA data portal (https://portal.gdc.cancer.gov/). Results shown here are based in part from data generated by the TCGA Research Network: http://cancergenome.nih.gov/ and the International Cancer Genome Consortium (ICGC): https://icgc.org/. The results presented in the current publication are based in part on the use of study data downloaded from the dbGaP web site.

Research involving human participants, their data, or biological material

Policy information about studies with human participants or human data. See also policy information about sex, gender (identity/presentation), and sexual orientation and race, ethnicity and racism.

Reporting on sex and gender	Sex and gender of the Dana-Farber cohort are included in Supplemental table. Comparative analyses were not performed due to lack of power for this analysis.
Reporting on race, ethnicity, or other socially relevant groupings	Race and ethnicity of the Dana-Farber cohort are included in Supplemental table. Comparative analyses were not performed due to lack of power for this analysis.
Population characteristics	The Dana-Farber cohort includes a selected population of patients with a median age of 62 years, predominantly male and white. Limited demographic data were available for this Caris cohort due to the deidentified nature of the cohort.
Recruitment	Dana-Farber cohort: Patients were included if they consented to the biobanking protocol 03-189 and they had been treated with a platinum containing agent. Caris cohort: All patients with gastroesophageal cancer who had sequencing analyses and Medicare claims deidentified data were included
Ethics oversight	The Internal Review Board of the Dana-Farber Cancer Institute, Boston, Massachusetts, USA (protocol 03-189). The remaining clinical analyses were IRB exempt.

Note that full information on the approval of the study protocol must also be provided in the manuscript.

Field-specific reporting

Please select the one below that is the best fit for your research. If you are not sure, read the appropriate sections before making your selection.

☒ Life sciences ☐ Behavioural & social sciences ☐ Ecological, evolutionary & environmental sciences

For a reference copy of the document with all sections, see nature.com/documents/nr-reporting-summary-flat.pdf

Life sciences study design

All studies must disclose on these points even when the disclosure is negative.

Sample size	For all in vitro experiments, at least three biological replicates were used.
Data exclusions	Patient samples were excluded if the number of SNV and short indel mutations were smaller than 50.
Replication	All data was reproduced using biological replicates and/or technical replicates. Each in vitro experiment was repeated at least once. Sample sizes were large enough to sure appropriate representation of the population behavior.
Randomization	N/A
Blinding	N/A

Reporting for specific materials, systems and methods

We require information from authors about some types of materials, experimental systems and methods used in many studies. Here, indicate whether each material, system or method listed is relevant to your study. If you are not sure if a list item applies to your research, read the appropriate section before selecting a response.

Materials & experimental systems

n/a	Involved in the study
☐	☒ Antibodies
☐	☒ Eukaryotic cell lines
☒	☐ Palaeontology and archaeology
☒	☐ Animals and other organisms
☐	☒ Clinical data
☒	☐ Dual use research of concern
☒	☐ Plants

Methods

n/a	Involved in the study
☒	☐ ChIP-seq
☒	☐ Flow cytometry
☒	☐ MRI-based neuroimaging

Antibodies

Antibodies used: TotalSeqTM-C0251 Hashtag 1 (BioLegend #394661) and TotalSeqTM-C0252 Hashtag 2 (BioLegend #394662), rabbit anti-HA antibody (1:600; CST #3724S), goat anti-rabbit antibody coupled to Alexa fluor488 fluorochrome (1:600; Lifetech #A11008),6-4PP primary antibody (Cosmo Bio #NM-DND-002, 1:2000), Rad51 Rabbit 1:200, CST # 8875 and pH2A.X Mouse 1:600 CST #80312, anti-Rabbit IgG Alexa Fluor 488 (Life tech #A11008) and anti-Mouse IgG Alexa Fluor 568 (Life tech #A11004),

Validation: All the antibodies are commercial (see above) and pre-validated by the respective manufacturers.

Eukaryotic cell lines

Policy information about cell lines and Sex and Gender in Research

Cell line source(s): Human gastric cancer cell lines were obtained from the Cancer Cell Line Encyclopedia (CCLE) core facility (BROAD Institute, Cambridge), which obtained them directly from commercial sources and authenticated the lines using standard short tandem repeat analysis. RPE and MDA-MB-468 cells were obtained from ATCC. For more information please see "Cell lines and cell culture".

Authentication: see a above (short tandem repeat analysis)

Mycoplasma contamination: Mycoplasma testing was performed every month using MycoAlert® Mycoplasma Detection Kit (Lonza #LT07-118)and found to be negative on each check.

Commonly misidentified lines (See ICLAC register): None of the cell lines are in this register.

Clinical data

Policy information about clinical studies

All manuscripts should comply with the ICMJE guidelines for publication of clinical research and a completed CONSORT checklist must be included with all submissions.

Clinical trial registration: N/A

Study protocol: N/A

Data collection: N/A

Outcomes: N/A

Plants

Seed stocks: N/A

Novel plant genotypes: N/A

Authentication: N/A
