# Supplementary material for: Mutational signature-based identification of DNA repair deficient gastroesophageal adenocarcinomas for therapeutic targeting
Source: NPJ Precis Oncol. 2024 Apr 8;8:87. doi: 10.1038/s41698-024-00561-6 (PMC11001913; doi:10.1038/s41698-024-00561-6)
Supplement: Supplementary file 2 — Supplementary Material [file 41698_2024_561_MOESM2_ESM.pdf]

Mutational signature-based identification of DNA repair deficient gastroesophageal adenocarcinomas for therapeutic targeting

Aurel Prosz*, Pranshu Sahgal*, Brandon M. Huffman, Zsofia Sztupinszki, Clare X. Morris, David Chen, Judit Börcsök, Miklos Diossy, Viktoria Tisza, Sandor Spisak, Pornlada Likasitwatanakul, Orsolya Rusz, Istvan Csabai, Michael Cecchini, Yasmine Baca, Andrew Elliott, Peter Enzinger, Harshabad Singh, Jessalyn Ubellaker, Jean-Bernard Lazaro, James M. Cleary, Zoltan Szallasi#, Nilay S. Sethi#
*equal contribution
#corresponding author

INVENTORY

Supplemental Material contains 19 Figures and 5 Tables (2 in .xls format).

Supplemental Figure 1-8 is related to Figure 1.
Supplemental Figure 10-13 is related to Figure 2.
Supplemental Figure 14-16 is related to Figure 3.
Supplemental Figure 17 is related to Figure 4.
Supplemental Figure 18-19 is related to Figure 5.

Supplementary Figures

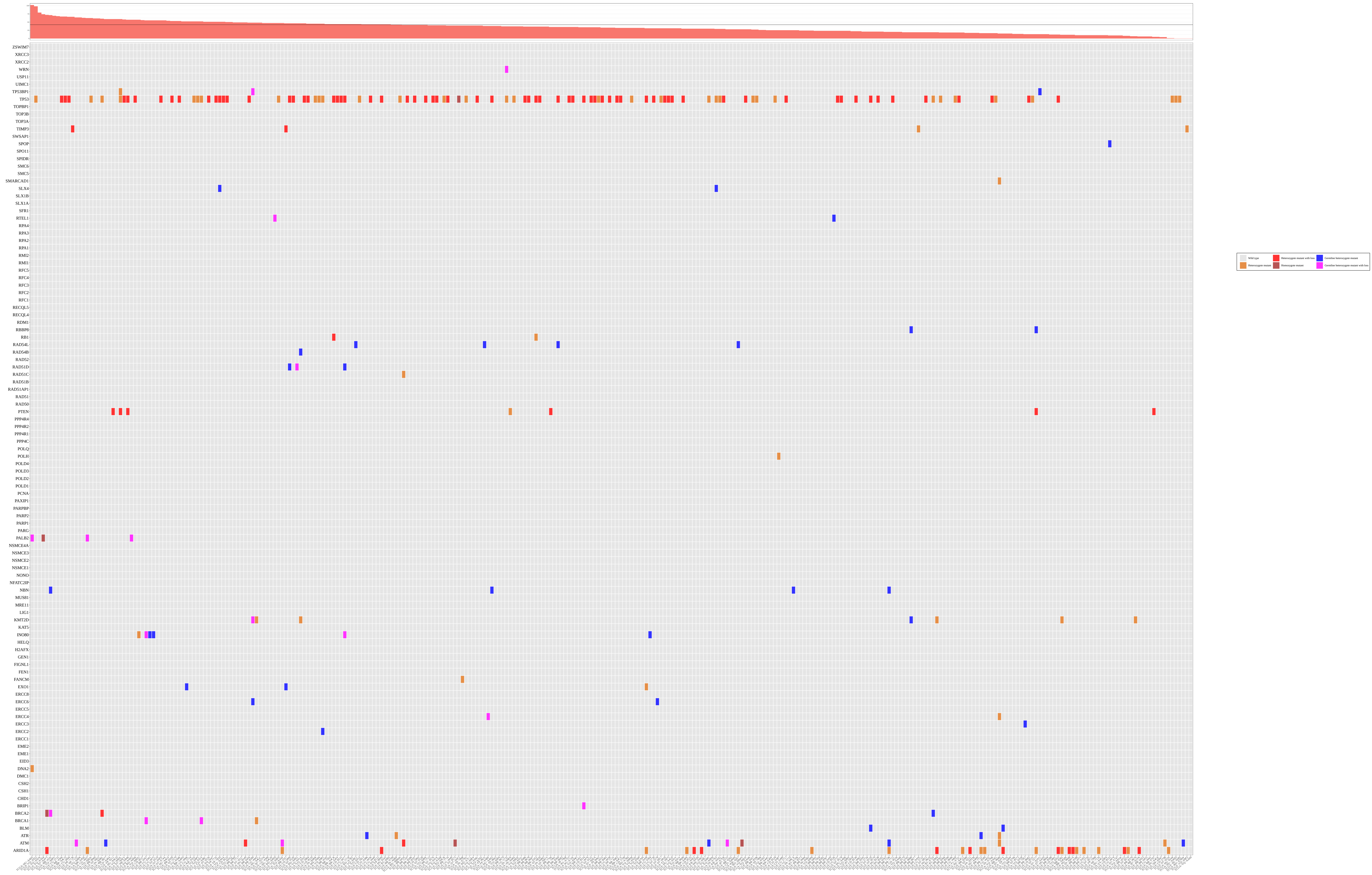
100
75
50
25
0
ZSWIM7
XRCC3
XRCC2
WRN
USP11
UIMC1
TP53BP1
TP53
TOPBP1
TOP3B
TOP3A
TIMP3
SWSAP1
SPOP
SPO11
SPIDR
SMC6
SMC5
SMARCAD1
SLX4
SLX1B
SLX1A
SFR1
RTEL1
RPA4
RPA3
RPA2
RPA1
RMI2
RMI1
RFC5
RFC4
RFC3
RFC2
RFC1
RECQL5
RECQL4
RDM1
RBBP8
RB1
RAD54L
RAD54B
RAD52
RAD51D
RAD51C
RAD51B
RAD51AP1
RAD51
RAD50
PTEN
PPP4R4
PPP4R2
PPP4R1
PPP4C
POLQ
POLH
POLD4
POLD3
POLD2
POLD1
PCNA
PAXIP1
PARPBP
PARP2
PARP1
PARG
PALB2
NSMCE4A
NSMCE3
NSMCE2
NSMCE1
NONO
NFATC2IP
NBN
MUS81
MRE11
LIG1
KMT2D
KAT5
INO80
HELQ
H2AFX
GEN1
FIGNL1
FEN1
FANCM
EXO1
ERCC8
ERCC6
ERCC5
ERCC4
ERCC3
ERCC2
ERCC1
EME2
EME1
EID3
DNA2
DMC1
CSH2
CSH1
CHD1
BRIP1
BRCA2
BRCA1
BLM
ATR
ATM
ARID1A

Supplemental Figure 1| Mutation status of HR genes and DNA damage/DNA-repair response in TCGA-STAD WES. Cases are ordered by their respective HRD scores.

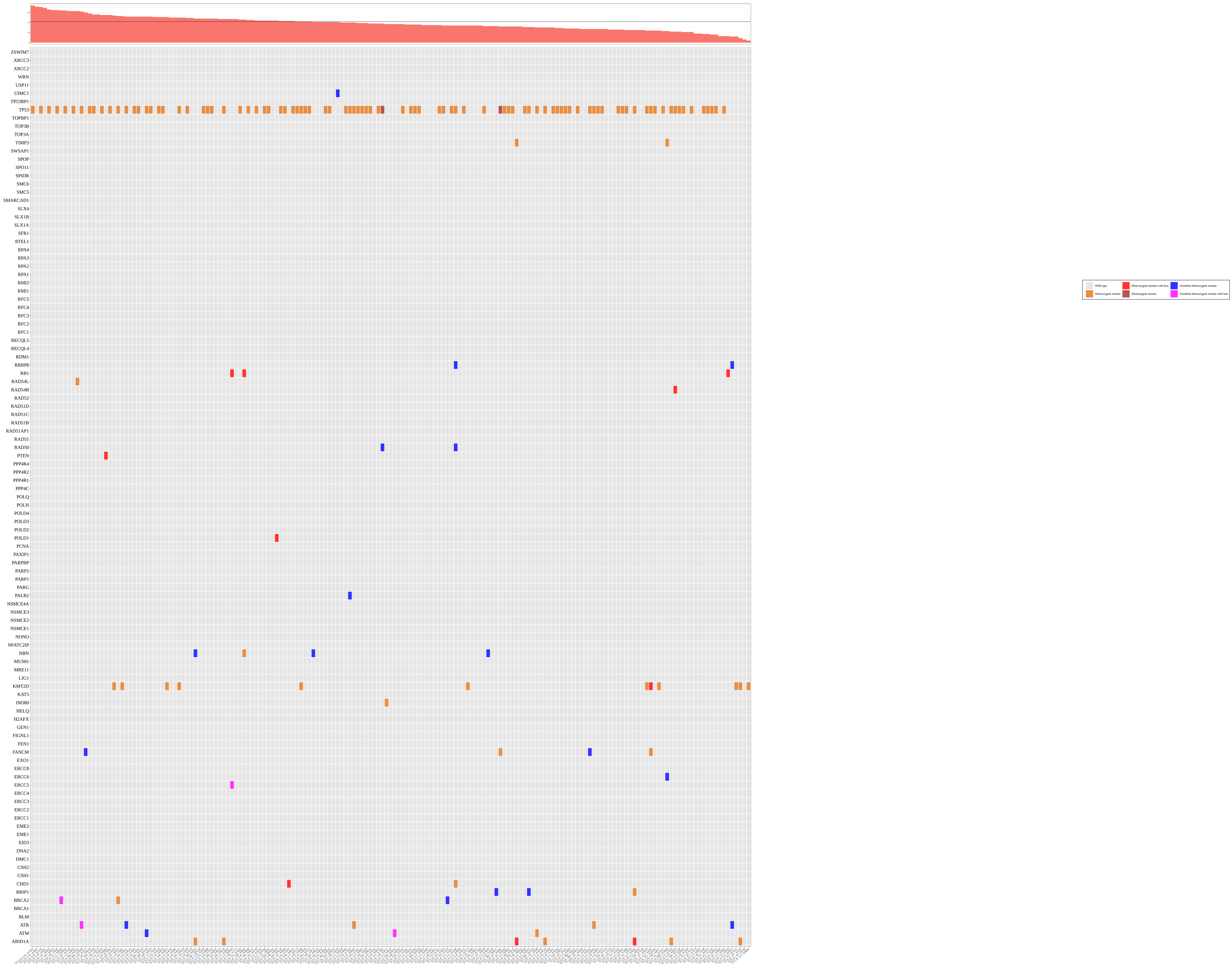

ZSWIM7
XRCC3
XRCC2
WRN
USP11
USMG1
TP53BP1
TP53
TOPBP1
TOP3B
TOP3A
TIMP3
SWSAP1
SPOP
SPO11
SPIDR
SMC6
SMC5
SMARCAD1
SLX4
SLX1B
SLX1A
SFR1
RTEL1
RPA4
RPA3
RPA2
RPA1
RMI2
RMI1
RFC5
RFC4
RFC3
RFC2
RFC1
RECQL5
RECQL4
RDM1
RBBP8
RB1
RAD54L
RAD54B
RAD52
RAD51D
RAD51C
RAD51B
RAD51AP1
RAD51
RAD50
PTEN
PPP4R4
PPP4R2
PPP4R1
PPP4C
POLQ
POLH
POLD4
POLD3
POLD2
POLD1
PCNA
PAXIP1
PARPBP
PARP2
PARP1
PARG
PALB2
NSMCE4A
NSMCE3
NSMCE2
NSMCE1
NONO
NFATC2IP
NBN
MUS81
MRE11
LIG1
KMT2D
KAT5
INO80
HELQ
H2AFX
GEN1
FIGNL1
FEN1
FANCM
EXO1
ERCC8
ERCC6
ERCC5
ERCC4
ERCC3
ERCC2
ERCC1
EME2
EME1
EID3
DNA2
DMC1
CSH2
CSH1
CHD1
BRIP1
BRCA2
BRCA1
BLM
ATR
ATM
ARID1A
Wild type
Heterozygote mutant with loss
Germline heterozygote mutant
Heterozygote mutant
Homozygote mutant
Germline heterozygote mutant with loss

Supplemental Figure 2| Mutation status of HR genes and DNA damage/DNA-repair response in TCGA-ESCA WES samples. Cases are ordered by their respective HRD scores.

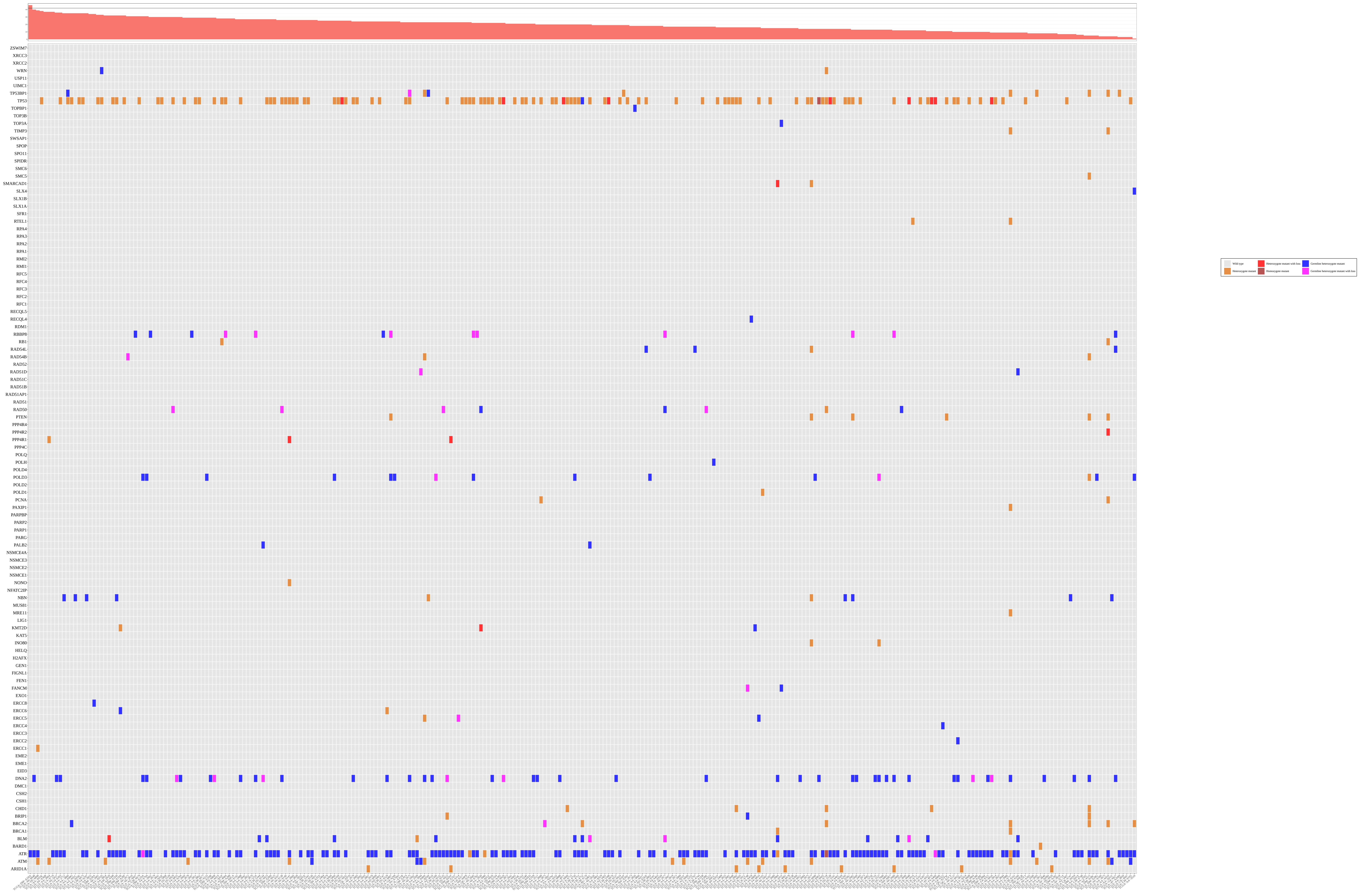
ZSWIM7
XRCC3
XRCC2
WRN
USP11
UIMC1
TP53BP1
TP53
TOPBP1
TOP3B
TOP3A
TIMP3
SWSAP1
SPOP
SPO11
SPIDR
SMC6
SMC5
SMARCAD1
SLX4
SLX1B
SLX1A
SFR1
RTEL1
RPA4
RPA3
RPA2
RPA1
RMI2
RMI1
RFC5
RFC4
RFC3
RFC2
RFC1
RECQL5
RECQL4
RDM1
RBBP8
RB1
RAD54L
RAD54B
RAD52
RAD51D
RAD51C
RAD51B
RAD51AP1
RAD51
RAD50
PTEN
PPP4R4
PPP4R2
PPP4R1
PPP4C
POLQ
POLH
POLD4
POLD3
POLD2
POLD1
PCNA
PAXIP1
PARPBP
PARP2
PARP1
PARG
PALB2
NSMCE4A
NSMCE3
NSMCE2
NSMCE1
NONO
NFATC2IP
NBN
MUS81
MRE11
LIG1
KMT2D
KAT5
INO80
HELQ
H2AFX
GEN1
FIGNL1
FEN1
FANCM
EXO1
ERCC8
ERCC6
ERCC5
ERCC4
ERCC3
ERCC2
ERCC1
EME2
EME1
EID3
DNA2
DMC1
CSH2
CSH1
CHD1
BRIP1
BRCA2
BRCA1
BLM
BARD1
ATR
ATM
ARID1A
Wild type
Heterozygote mutant
Heterozygote mutant with loss
Homozygote mutant
Germline heterozygote mutant
Germline heterozygote mutant with loss

Supplemental Figure 3| Mutation status of HR genes and DNA damage/DNA-repair response in TCGA-COAD WES samples. Cases are ordered by their respective HRD scores.

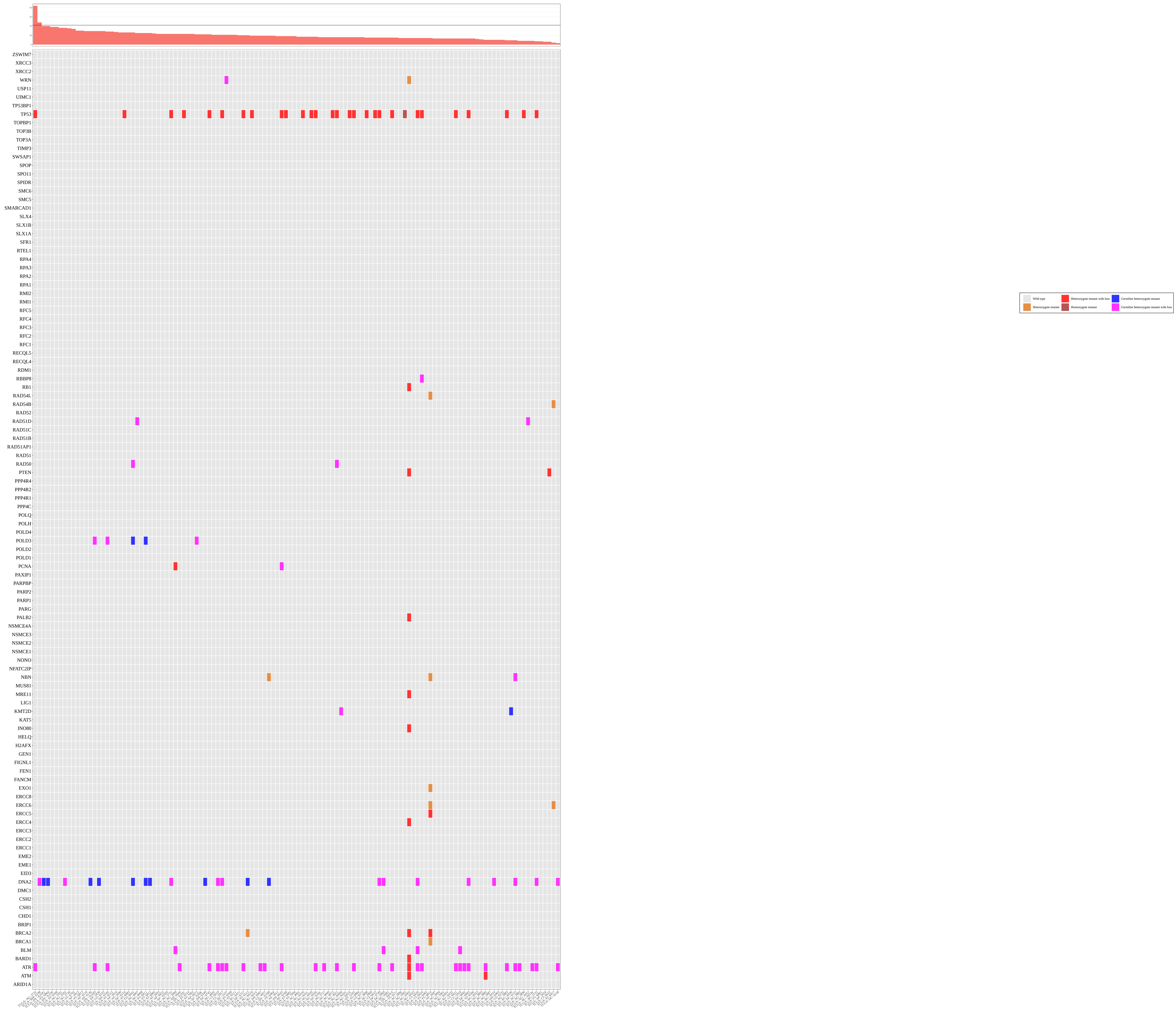
ZSWIM7
XRCC3
XRCC2
WRN
USP11
UIMC1
TP53BP1
TP53
TOPBP1
TOP3B
TOP3A
TIMP3
SWSAP1
SPOP
SPO11
SPIDR
SMC6
SMC5
SMARCAD1
SLX4
SLX1B
SLX1A
SFR1
RTEL1
RPA4
RPA3
RPA2
RPA1
RMI2
RMI1
RFC5
RFC4
RFC3
RFC2
RFC1
RECQL5
RECQL4
RDM1
RBBP8
RB1
RAD54L
RAD54B
RAD52
RAD51D
RAD51C
RAD51B
RAD51AP1
RAD51
RAD50
PTEN
PPP4R4
PPP4R2
PPP4R1
PPP4C
POLQ
POLH
POLD4
POLD3
POLD2
POLD1
PCNA
PAXIP1
PARPBP
PARP2
PARP1
PARG
PALB2
NSMCE4A
NSMCE3
NSMCE2
NSMCE1
NONO
NFATC2IP
NBN
MUS81
MRE11
LIG1
KMT2D
KAT5
INO80
HELQ
H2AFX
GEN1
FIGNL1
FEN1
FANCM
EXO1
ERCC8
ERCC6
ERCC5
ERCC4
ERCC3
ERCC2
ERCC1
EME2
EME1
EID3
DNA2
DMC1
CSH2
CSH1
CHD1
BRIP1
BRCA2
BRCA1
BLM
BARD1
ATR
ATM
ARID1A
Wild type
Heterozygote mutant with loss
Germline heterozygote mutant
Heterozygote mutant
Homozygote mutant
Germline heterozygote mutant with loss

Supplemental Figure 4| Mutation status of HR genes and DNA damage/DNA-repair response in TCGA-READ WES samples. Cases are ordered by their respective HRD scores.

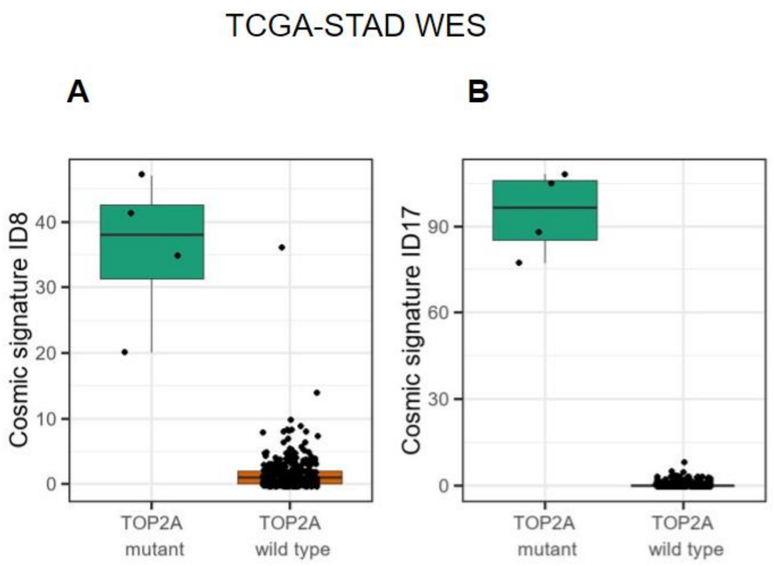

Supplemental Figure 5| Cosmic signature ID8 and ID17 in the TCGA-STAD WES cohort compared between TOP2A mutants and WT cases. P-values calculated by Mann-Whitney test.

A. Distribution of Cosmic signature ID8 absolute counts.
B. Distribution of Cosmic signature ID17 absolute counts.

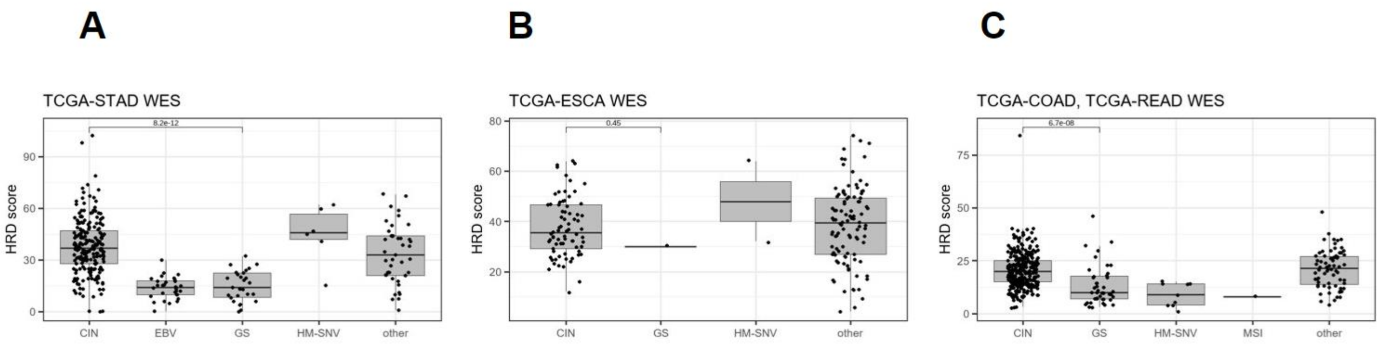

Supplemental Figure 6| HRD score compared across different molecular subtypes in the TCGA-STAD WES (A), TCGA-ESCA WES (B), TCGA-COAD/READ WES (C) cohorts. The subtypes are: chromosomal instability (CIN), Epstein–Barr virus (EBV), genomically stable (GS), hypermutated-SNV (HM-SNV). The category other refers to the samples which could not be classified. P-values calculated by Mann Whitney test between the chromosomal instability and genomically stable subtypes.

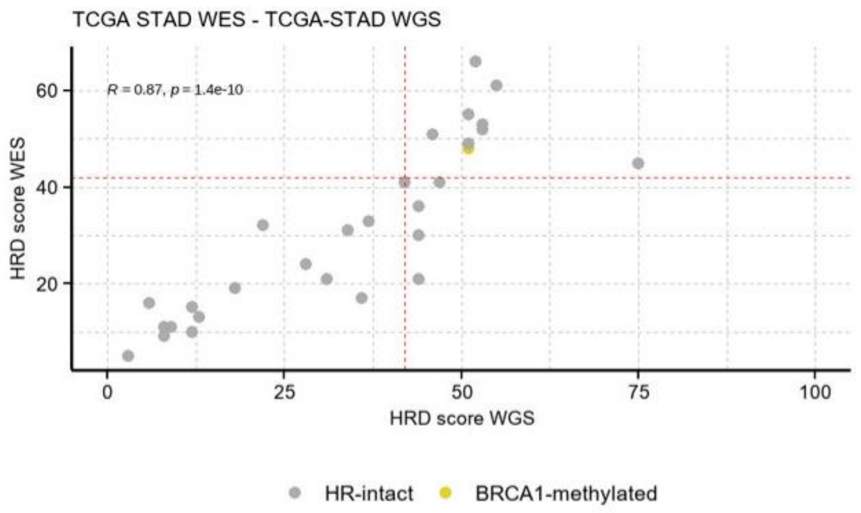

Supplemental Figure 7| Correlation between the TCGA-STAD WES and TCGA-STAD WGS HRD scores, calculated for the overlapping samples. Pearson correlation coefficient (0.87), and p-value (1.4e-10) were calculated between the HRD scores of the same TCGA-STAD WES and TCGA-STAD WGS samples and the one BRCA1-methylated case is highlighted.

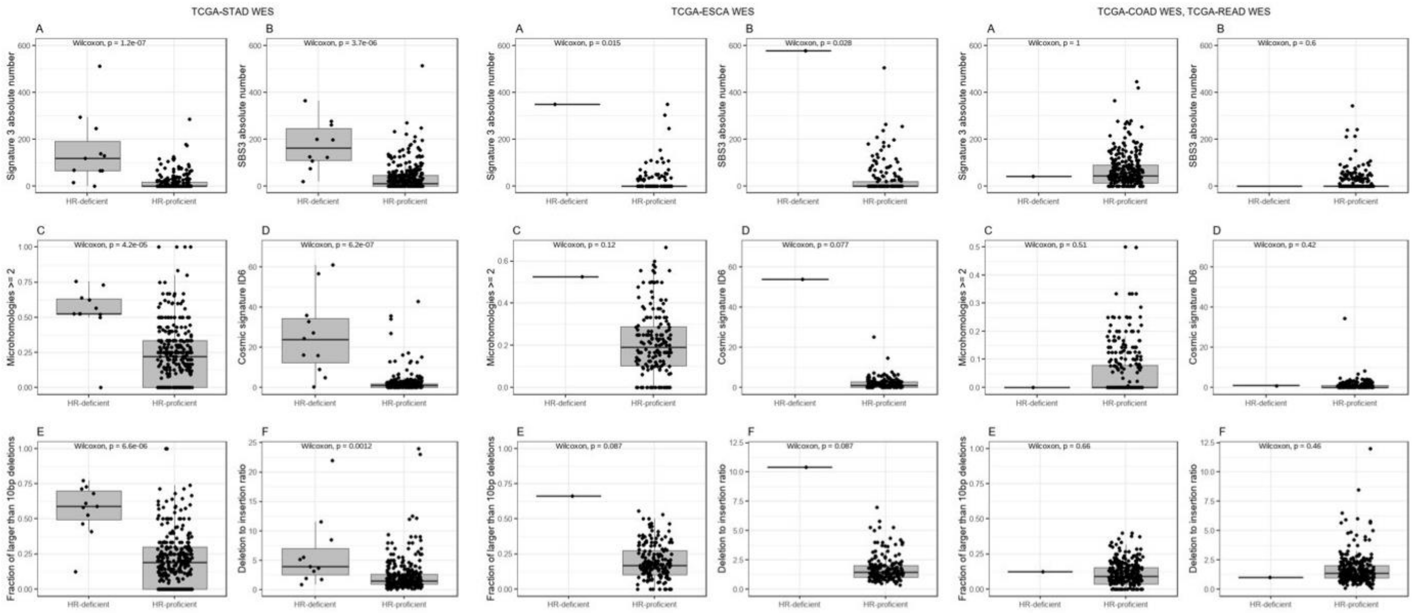

Supplemental Figure 8| Distribution of signatures associated with BRCA1/2 deficiency in the TCGA STAD WES, TCGA ESCA WES and TCGA COAD/READ WES cohorts. P-values calculated by Mann Whitney test.

A. Cosmic V2 signature 3 absolute counts.
B. Cosmic V3 SBS3 absolute counts.
C. Fraction of microhomologies at indels, which are equal or longer than 2 base-pairs in length.
D. Cosmic signature ID6 absolute counts.
E. Fraction of larger than 10 base-pair deletions.
F. Deletions to insertion ratio.

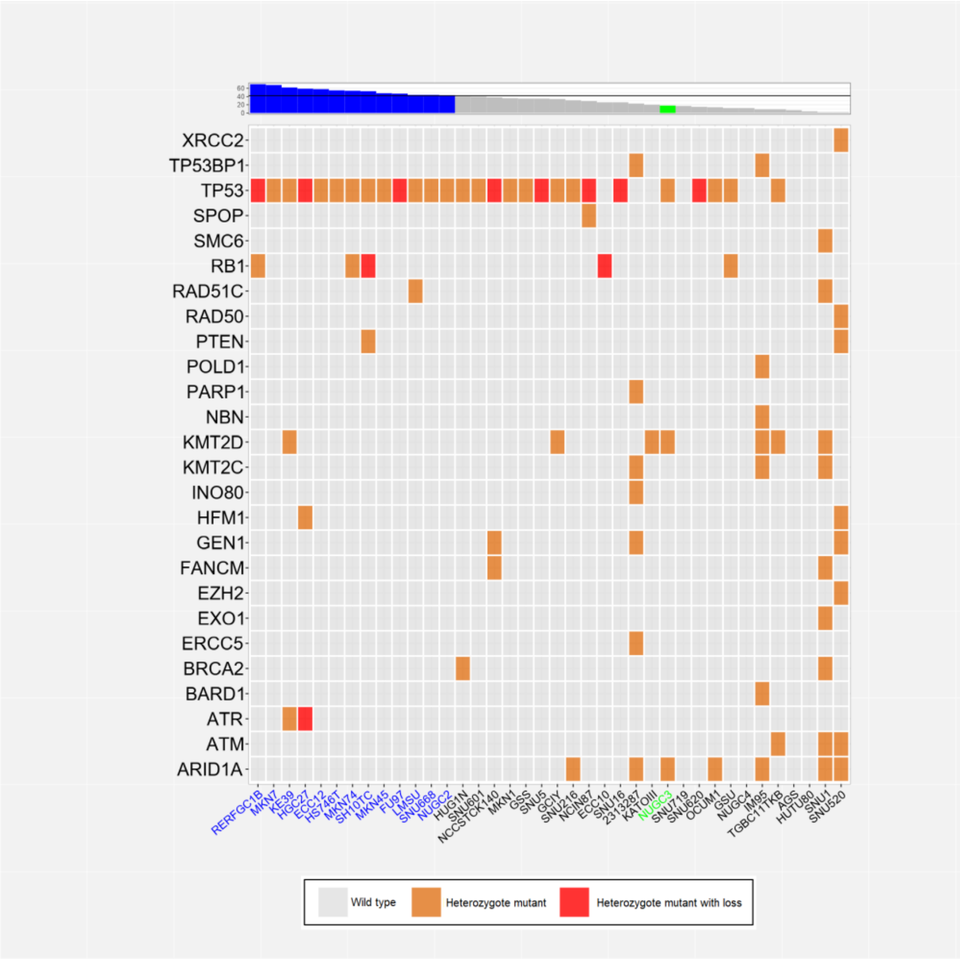

Supplemental Figure 9| Mutation status of HR genes and DNA damage/DNA-repair response in gastric cancer and non-neoplastic cell lines. Cell lines are ordered by their respective HRD scores, and they are highlighted in blue, if the HRD score is higher than 42, and NUGC3 is highlighted in green, indicating the functional NER deficiency.

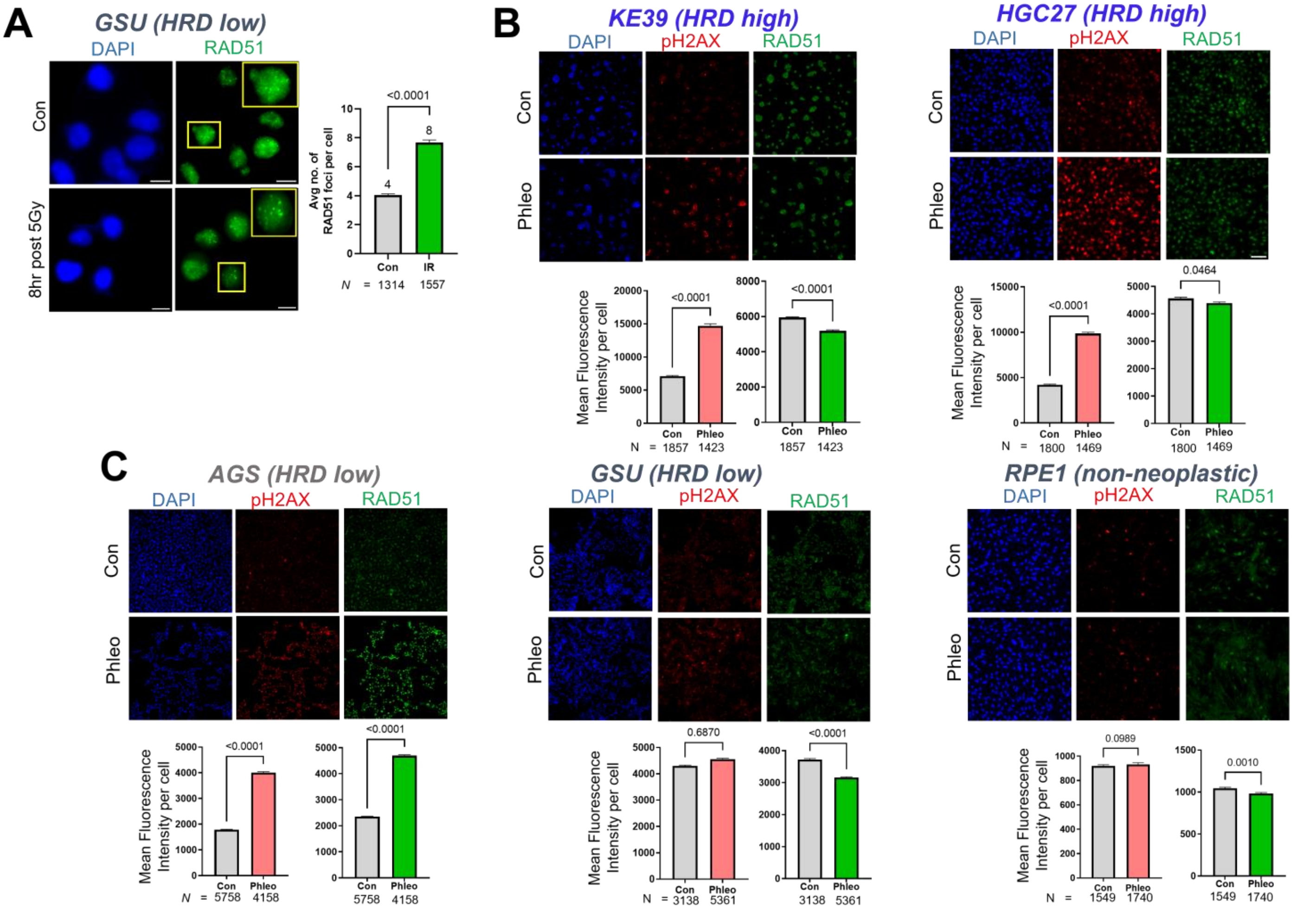

Supplemental Figure 10| Mutation status of HR genes and DNA damage/DNA-repair response in gastric cancer and non-neoplastic cell lines

A. Representative immunofluorescence images (left) and quantification (right) for GSU cells; Number of RAD51 (green) positive foci indicating DNA repair after treatment with 5Gy gamma radiation; Data expressed as the mean number of RAD51 positive foci per cell ± SEM; Scale bar=10um.

B. Representative immunofluorescence images and quantification from of pH2AX signal (red) indicating DS-DNA breaks and RAD51 signal (green) indicating DS-DNA repair after treatment with DMSO control or radiomemetic (gamma ray) phleomycin in KE39 and HGC27 cell lines; Data expressed as mean fluorescence intensity per cell ± SEM from the number of cells mentioned in the graph for each condition. P-values calculated by Mann Whitney test; Scale bar=100um.

C. Representative immunofluorescence images and quantification from of pH2AX signal (red) indicating DS-DNA breaks and RAD51 signal (green) indicating DS-DNA repair after treatment with DMSO control or radiomemetic (gamma ray) phleomycin in AGS, GSU and RPE1 cell lines; Data expressed as mean fluorescence intensity per cell ± SEM from the number of cells mentioned in the graph for each condition. P-values calculated by Mann Whitney test; Scale bar=100um.

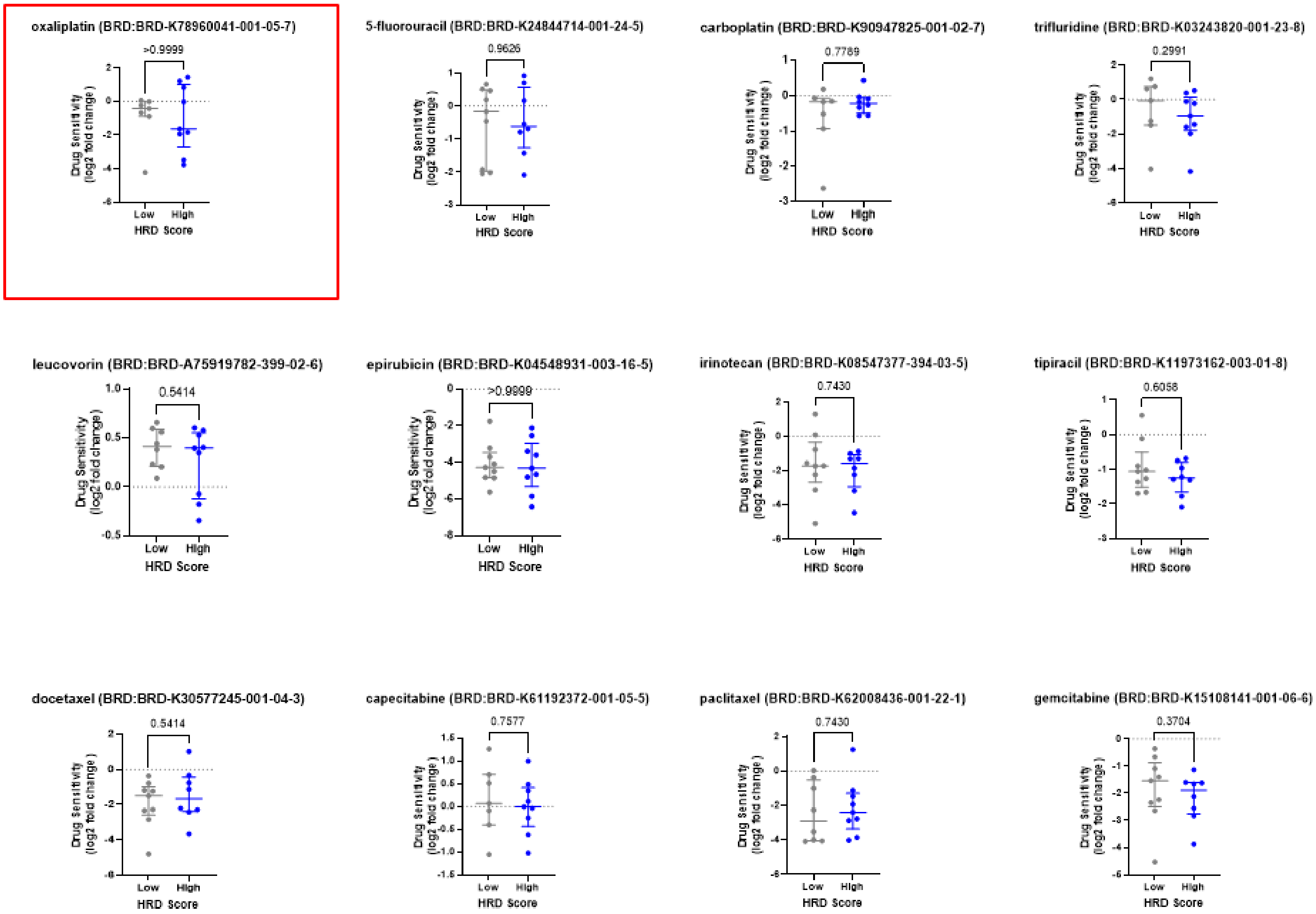

Supplemental Figure 11| Differential sensitivity of Low (grey) and high (blue) HRD score groups of gastric cancer cell lines for chemotherapeutic agents in BROAD institute PRISM repurposing drug screen dataset

Commonly used chemotherapeutic agents in GI cancers and other cancers treatment regimen. Difference between the drug sensitivity (log2 fold change) is represented as the median. P-values calculated by Mann Whitney test.

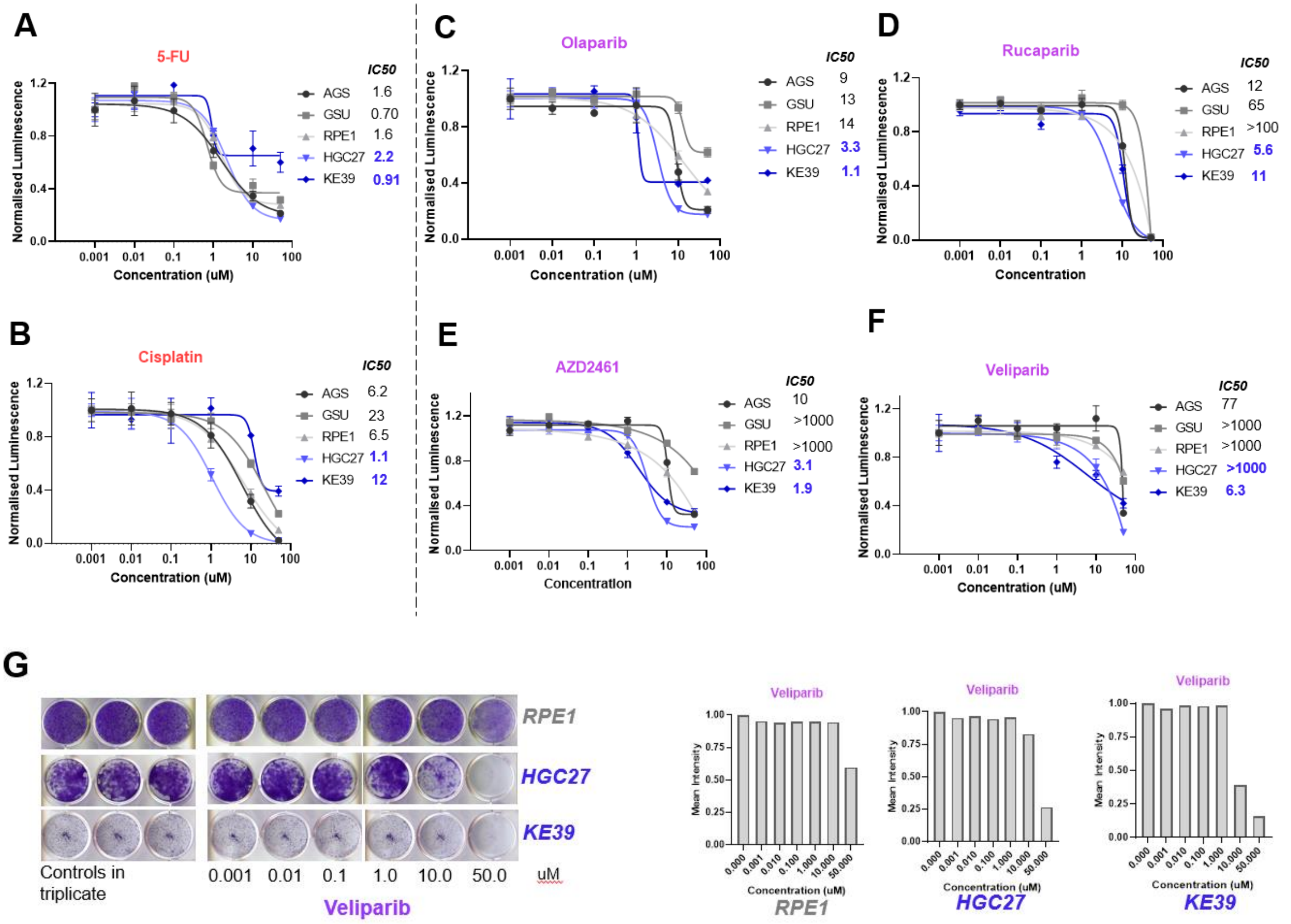

Supplemental Figure 12| Differential sensitivity of Low (grey) and high (blue) HRD score gastric cell lines for chemotherapeutic agents (red) and PARP inhibitors (magenta)

A. Dose-response curve of non-neoplastic cell line RPE1, Low HRD (grey) and high HRD (blue) score gastric cancer cell lines to indicated concentrations of 5-FU (red). Best-fit IC50 scores are displayed. Data presented as mean ± SD of three culture replicates at each indicated dose.

B. Dose-response curve of non-neoplastic cell line RPE1, Low HRD (grey) and high HRD (blue) score gastric cancer cell lines to indicated concentrations of Cisplatin. Best-fit IC50 scores are displayed. Data presented as mean ± SD of three culture replicates at each indicated dose.

C. Dose-response curve of non-neoplastic cell line RPE1, Low HRD (grey) and high HRD (blue) score gastric cancer cell lines to indicated concentrations of Olaparib (magenta). Best-fit IC50 scores are displayed. Data presented as mean ± SD of three culture replicates at each indicated dose.

D. Dose-response curve of non-neoplastic cell line RPE1, Low HRD (grey) and high HRD (blue) score gastric cancer cell lines to indicated concentrations of Rucaparib (magenta). Best-fit IC50 scores are displayed. Data presented as mean ± SD of three culture replicates at each indicated dose.

E. Dose-response curve of non-neoplastic cell line RPE1, Low HRD (grey) and high HRD (blue) score gastric cancer cell lines to indicated concentrations of AZD2462 (magenta). Best-fit IC50 scores are displayed. Data presented as mean ± SD of three culture replicates at each indicated dose.

F. Dose-response curve of non-neoplastic cell line RPE1, Low HRD (grey) and high HRD (blue) score gastric cancer cell lines to indicated concentrations of Veliparib (magenta). Best-fit IC50 scores are displayed. Data presented as mean ± SD of three culture replicates at each indicated dose.

G. Colony formation assay (left) and the quantification shown as mean intensity from each well using imageJ (right) of gastric cancer cell with High HRD score (in blue font) and non-neoplastic RPE1 cell line (in grey).

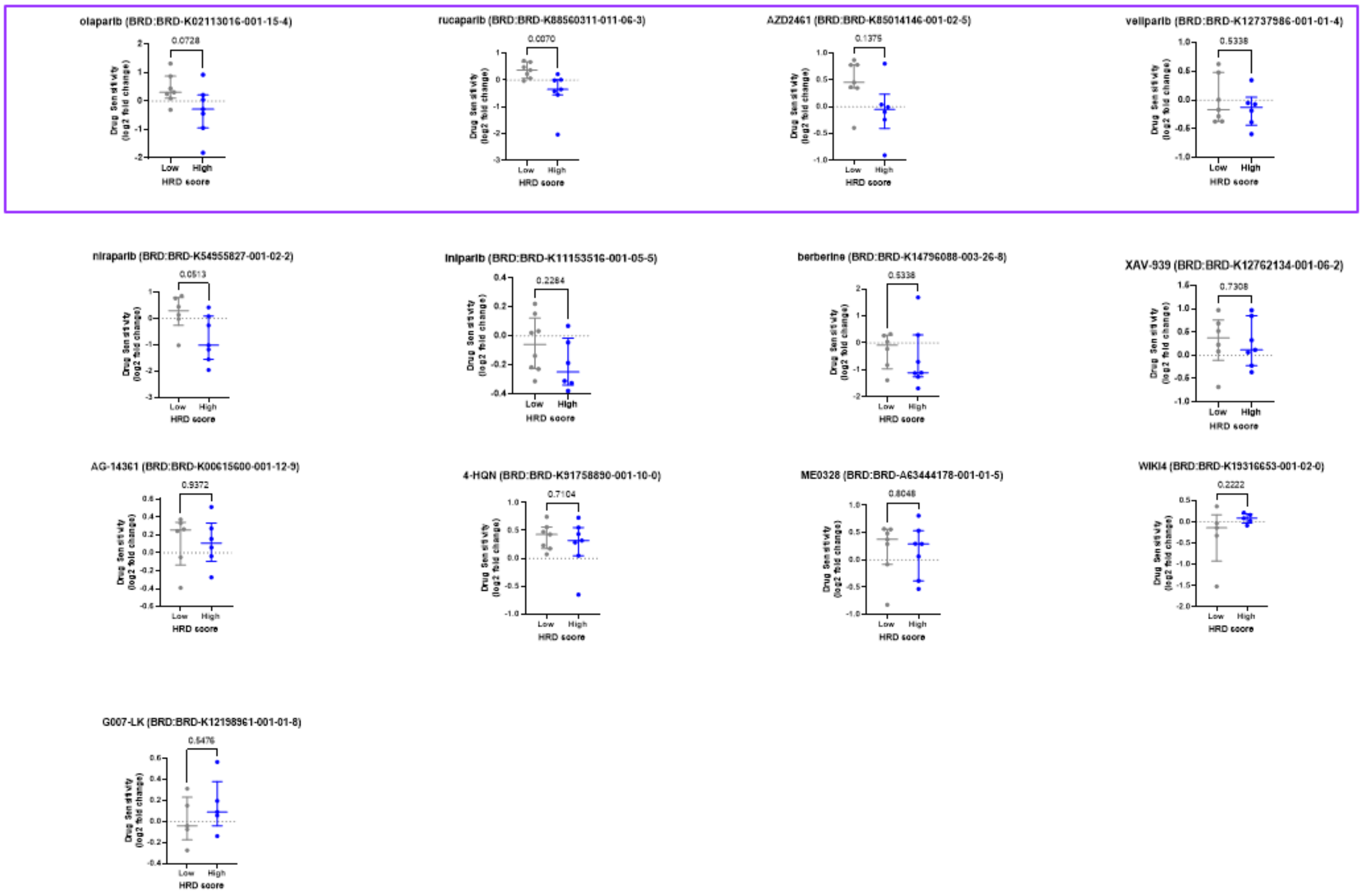

Supplemental Figure 13| Differential sensitivity of Low (grey) and high (blue) HRD score groups of gastric cancer cell lines for PARP inhibitors in BROAD institute PRISM repurposing drug screen dataset

Commonly used PARP inhibitors for different cancers. Difference between the drug sensitivity (log2 fold change) is represented as the median. P-values calculated by Mann Whitney test.

PARP inhibitors used in this study are enclosed in magenta box (along with the talazoparib shown in the main figure).

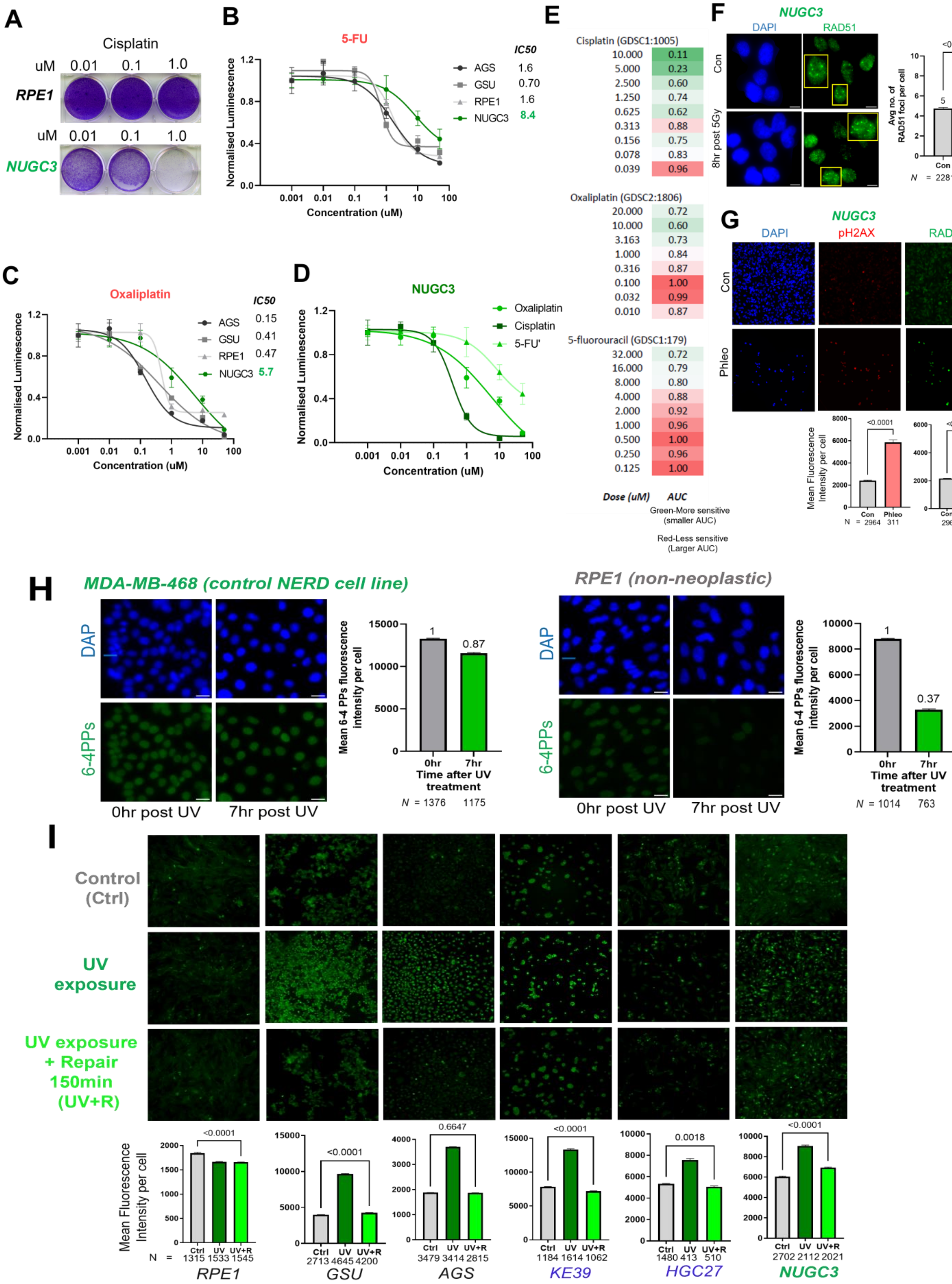
A
Cisplatin
uM 0.01 0.1 1.0
RPE1
uM 0.01 0.1 1.0
NUGC3
B
5-FU
IC50
AGS 1.6
GSU 0.70
RPE1 1.6
NUGC3 8.4
Normalised Luminescence
Concentration (uM)
C
Oxaliplatin
IC50
AGS 0.15
GSU 0.41
RPE1 0.47
NUGC3 5.7
Normalised Luminescence
Concentration (uM)
D
NUGC3
Oxaliplatin
Cisplatin
5-FU'
Normalised Luminescence
Concentration (uM)
E
Cisplatin (GDSC1:1005)
10.000 0.11
5.000 0.23
2.500 0.60
1.250 0.74
0.625 0.62
0.313 0.88
0.156 0.75
0.078 0.83
0.039 0.96
Oxaliplatin (GDSC2:1806)
20.000 0.72
10.000 0.60
3.163 0.73
1.000 0.84
0.316 0.87
0.100 1.00
0.032 0.99
0.010 0.87
5-fluorouracil (GDSC1:179)
32.000 0.72
16.000 0.79
8.000 0.80
4.000 0.88
2.000 0.92
1.000 0.96
0.500 1.00
0.250 0.96
0.125 1.00
Dose (uM) AUC
Green-More sensitive (smaller AUC)
Red-Less sensitive (Larger AUC)
F
NUGC3
DAPI RAD51
Con
8hr post 5Gy
Avg no. of RAD51 foci per cell
<0.0001
5 8
Con IR
N = 2281 1569
G
NUGC3
DAPI pH2AX RAD51
Con
Phleo
Mean Fluorescence Intensity per cell
<0.0001 <0.0001
Con Phleo Con Phleo
N = 2964 311 2964 311
H
MDA-MB-468 (control NERD cell line)
DAP
6-4PPs
0hr post UV 7hr post UV
Mean 6-4 PPs fluorescence intensity per cell
1 0.87
0hr 7hr
Time after UV treatment
N = 1376 1175
RPE1 (non-neoplastic)
DAP
6-4PPs
0hr post UV 7hr post UV
Mean 6-4 PPs fluorescence intensity per cell
1 0.37
0hr 7hr
Time after UV treatment
N = 1014 763
I
Control (Ctrl)
UV exposure
UV exposure + Repair 150min (UV+R)
Mean Fluorescence Intensity per cell
<0.0001 <0.0001 0.6647 <0.0001 0.0018 <0.0001
Ctrl UV UV+R
N = 1315 1533 1545 RPE1
2713 4645 4200 GSU
3479 3414 2815 AGS
1184 1614 1062 KE39
1480 413 510 HGC27
2702 2112 2021 NUGC3
UV+R > Ctrl = True NER deficient

Supplemental Figure 14| Differential sensitivity of NUGC3 for chemotherapeutic agents

A. Colony formation assay showing the sensitivity of NUGC3 and RPE1 cell lines for cisplatin at three doses.

B. Dose-response curve of NUGC3 (green) and three control cell lines with intact DNA-repair pathways (grey) to indicated concentrations of chemotherapeutic agent 5-Fu. Best-fit IC50 scores are displayed. Data presented as mean ± SD. of three culture replicates at each indicated dose.

C. Dose-response curve of NUGC3 (green) and three control cell lines with intact DNA-repair pathways (grey) to indicated concentrations of chemotherapeutic agent Oxaliplatin Best-fit IC50 scores are displayed. Data presented as mean ± SD of three culture replicates at each indicated dose.

D. Dose-response curve of NUGC3 cell line to indicated concentrations of chemotherapeutic agents (Oxaliplatin, Cisplatin and 5-Fu). Best-fit IC50 scores are displayed. Data presented as mean ± SD of three culture replicates at each indicated dose.

E. NUGC3 cell line checked for Cisplatin, oxaliplatin, and 5-FU sensitivity in Sanger's Genomics of Drug Sensitivity in Cancer Project (GDSC1); Drug response measured as Area Under the Curve (AUC). Dark green color in the heatmap indicates smaller AUC hence higher sensitivity; dark red color indicates larger AUC and relatively lower sensitivity for the chemotherapeutic agent.

F. Representative immunofluorescence images (left) and quantification (right) for NUGC3 cells; Number of RAD51 (green) positive foci indicating DNA repair after treatment with 5Gy gamma radiation; Data expressed as the mean number of RAD51 positive foci per cell ± SEM; Scale bar=20um.

G. Representative immunofluorescence images (top) and quantification (bottom) of pH2AX (red) and RAD51 (green) in NUGC3 cells after treatment with radiomemetic (gamma ray) phleomycin. Data expressed as mean fluorescence intensity per cell ± SEM from the number of cells mentioned in the graph for each condition. P-values calculated by Mann Whitney test; Scale bar=100um.

H. Representative immunofluorescence images (left) and quantification (right) of 6-4 photoproducts (6-4PPs- green), monitoring the cells' ability to remove 6-4-photoproducts and assessing NER. NER activity is expressed by the loss of 6-4PPs signal post 7 hours of 40J/m^2 UV irradiation. MDA-MB-468 (Known NERD cell lines) and RPE1 (non-neoplastic cell line); Data expressed as mean ± SEM; Scale bar= 20um.

I. Representative immunofluorescence images (top) and quantification (bottom) for the DDB2 proteo-probe assay to detect GG-NER defects. DDB2 signal (green) indicates DNA damage after UV treatment (dark green bars). In cells with intact GG-NER, DDB2 signal reduces to the signal in the control cells (grey bars) by 150 minutes (light green bars), indicating repair. In cells lacking NER, the signal does not completely reduce to that in control cells (UV+R > Ctrl = True NER deficient); control (gray), HR-deficient (blue), and NER-deficient (green) cell lines. Data expressed as mean fluorescence intensity per cell ± SEM from the number of cells mentioned in the graph for each condition. P-values calculated by Mann Whitney test; Scale bar=100um.

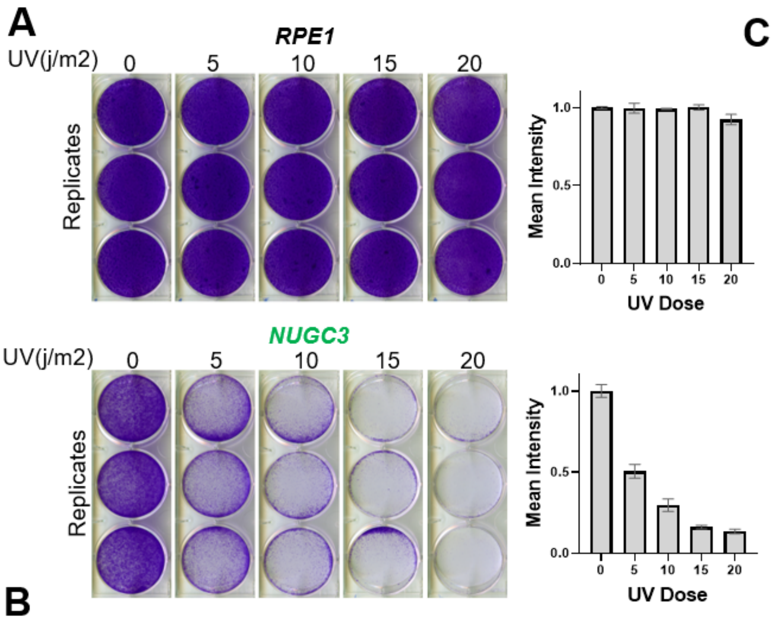

B

NUGC3

Gene	Variant classification	Variant annotation	Variant type	Protein change
ERCC6	Missense_Mutation	other non-conserving	SNP	p.D475Y
GTF2H1	Splice_Site	damaging	SNP	NA
TCEA1	In_Frame_Del	other non-conserving	DEL	p.K19del
CHD1L#	Missense_Mutation	other non-conserving	SNP	p.R633Q
XPC	Missense_Mutation	other non-conserving	SNP	p.S225N
MCRS1	Splice_Site	damaging	SNP	p.Q149H
POLE	Nonsense_Mutation	damaging	SNP	p.W1348*
RPA1	Frame_Shift_Del	damaging	DEL	p.G179fs

TCR-NER genes
GG-NER genes
Shared-NER genes

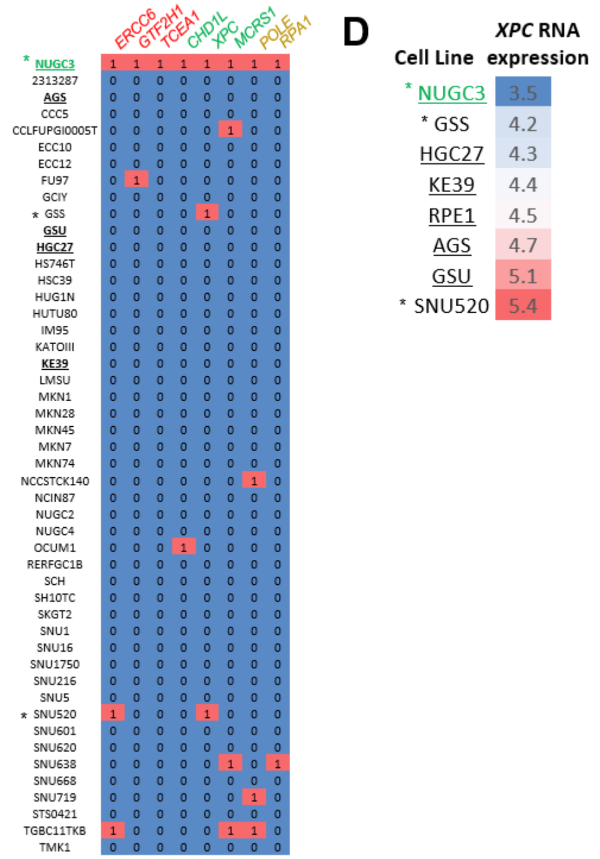

Supplemental Figure 15 | UV-C sensitivity and mutation profile of important NER genes in NUGC3 cell line.

A. Colony formation assay showing the UV radiation dose-dependent sensitivity (left) and quantification (right) of NUGC3 and RPE1 cell lines. Data presented as mean intensity ± SD of three culture replicates at each indicated UV dose.

B. Somatic point mutations and indels in important NER pathway genes in NUGC3 cell line. TCR-NER genes in red, GG-NER genes in green, Shared-NER genes in yellow. Data source: DepMap Internal 22Q1 mutations. #Known pathogenic mutation in breast cancer (COSMIC Genomic Mutation ID COSV63615250).

C. Presence (red/1) or absence (blue/0) of somatic point mutations and indels in important NER pathway genes in all the CCLE gastric cancer cell lines. TCR-NER genes in red, GG-NER genes in green, Shared-NER genes in yellow; NUGC3 in green, Cell lines used in this study are underlined, cell lines with xpc mutation are marked by a star. Data source: DepMap Internal 22Q1 mutations.

D. RNA *XPC* expression TPM values. Dark blue color in the heatmap indicates the lowest expression of xpc; dark red color indicates relatively highest expression of xpc; NUGC3 in green, Cell lines used in this study are underlined, Cell lines with *XPC* mutation are marked by a star. Data source: DepMap Expression 22Q1 Internal.

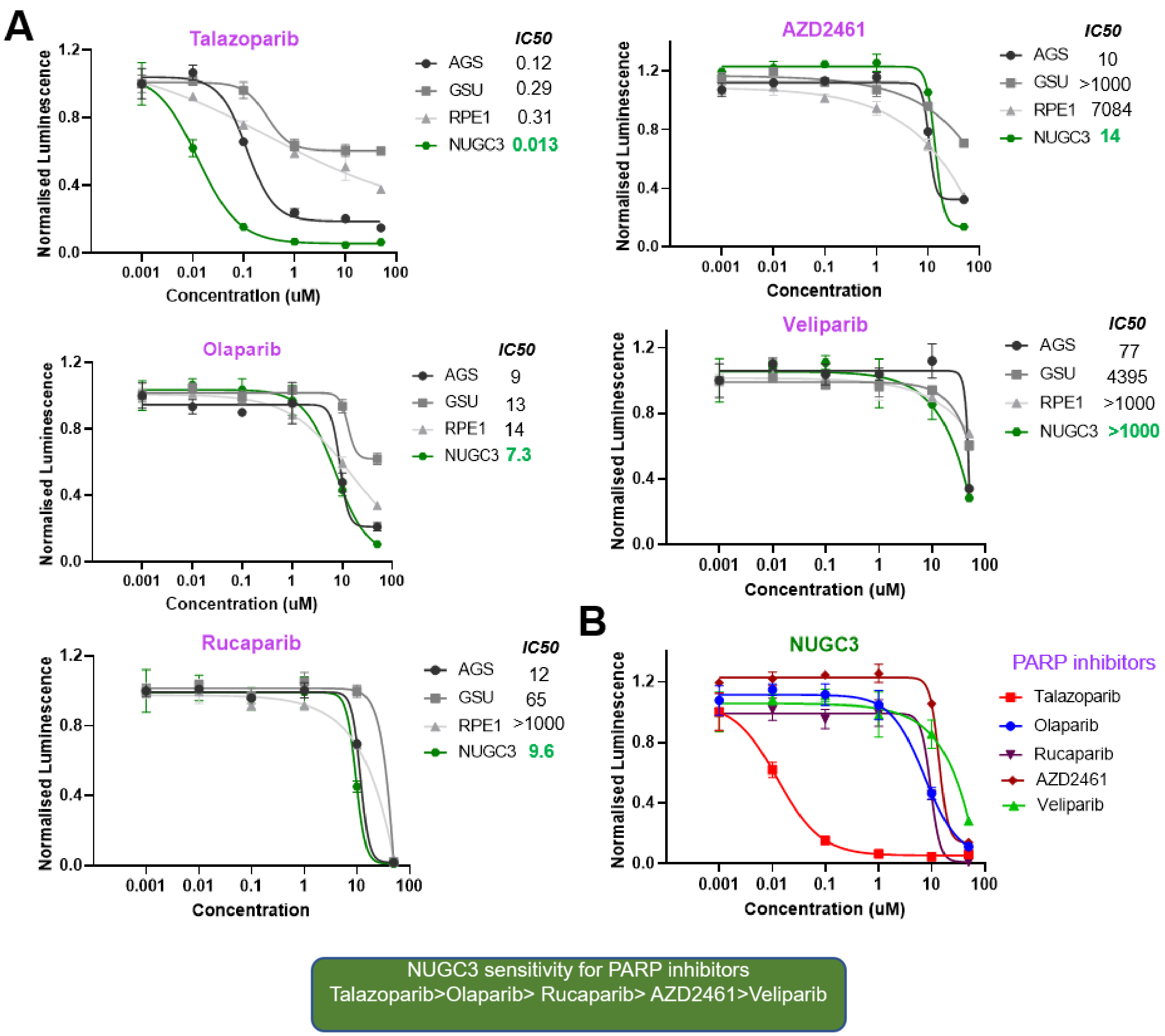

Supplemental Figure 16| Differential sensitivity of NUGC3 for PARP inhibitors

A. Dose-response curve of NUGC3 (green) and three control cell lines with intact DNA-repair pathways (grey) to indicated concentrations of PARP inhibitors (Talazoparib, Olaparib, Rucaparib, AZD2461, and Veliparib). Best-fit IC50 scores are displayed. Data presented as mean ± SD of three culture replicates at each indicated dose.

B. Dose-response curve of NUGC3 cell line to indicated concentrations of PARP inhibitors (Talazoparib, Olaparib, Rucaparib, AZD2461 and Veliparib). Best-fit IC50 scores are displayed. Data presented as mean ± SD of three culture replicates at each indicated dose.

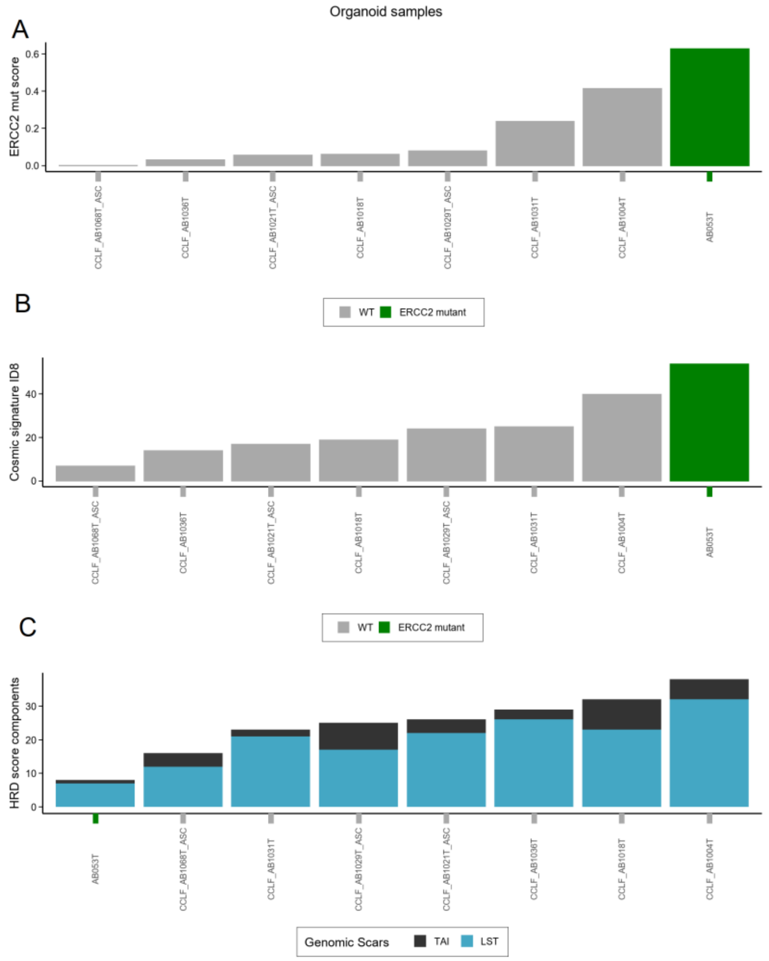

Supplemental Figure 17| The NER deficiency associated mutational signature score (ERCC2 mut score), Cosmic signature ID8, and two of the HRD score components in the organoid samples

A. Organoid samples ordered by ERCC2 mut score. The ERCC2 mutant sample is also highlighted here.
B. Organoid samples ordered by Cosmic signature ID8 absolute counts, which is also associated with NER deficiency.
C. Organoid samples ordered by the sum of two of the HRD score components. HRD-LOH was not calculated here, since the normal samples for the organoids were not available.

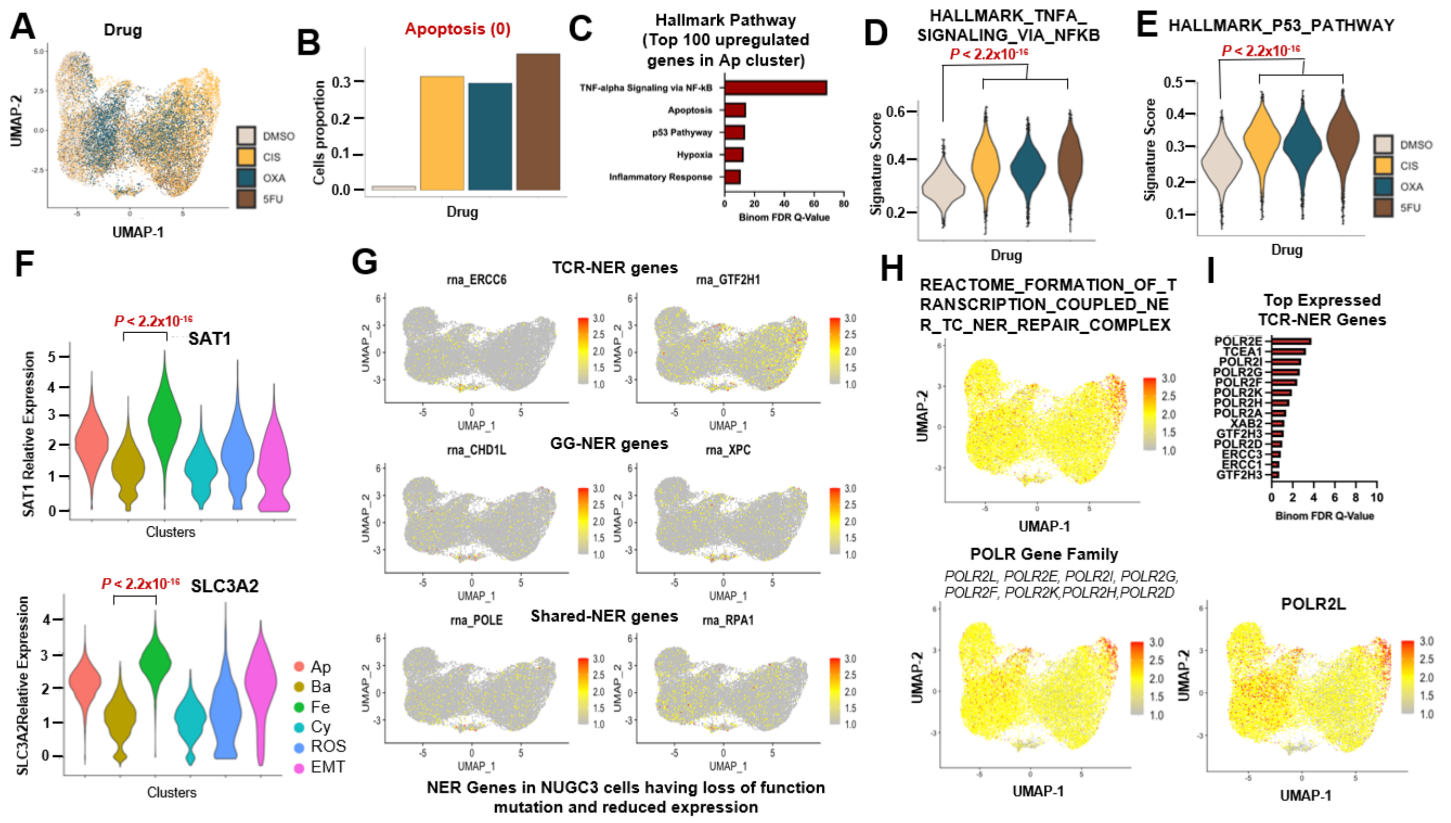

Supplemental Figure 18 | scRNA-seq analysis of NER deficient gastric cancer cells

A. UMAP representation of single cell transcriptome profiling of NUGC3 cells treated with DMSO (control) and Cisplatin, Oxaliplatin or 5FU (experimental) colored by and four treatment groups/sample identity.

B. Normalized cell counts in each treatment group for Apoptosis (0) cluster.

C. Hallmark pathways enrichment analysis (EnrichR) of top 100 genes upregulated in Apoptosis (0).

D. Violin plots indicating expression of HALLMARK_TNFA_SIGNALING_VIA_NFKB pathway in the four treatment groups revealed by hallmark pathways enrichment analysis (EnrichR) of top genes upregulated in cluster Ap(0) (also Supp table 1) . P-value calculated by Wilcoxon rank sum test with Bonferroni correction.

E. Violin plots indicating expression of HALLMARK_P53 pathway in the four treatment groups revealed by hallmark pathways enrichment analysis (EnrichR) of top genes upregulated in cluster Ap(0) (also Supp table 1) . P-value calculated by Wilcoxon rank sum test with Bonferroni correction.

F. Violin plots indicating SAT1 (top) and SLC3A2 (bottom) gene expression in different cell clusters. P-value calculated by Wilcoxon rank sum test with Bonferroni correction.

G. UMAP representation of genes mutated in three subcategory of NER pathways in NUGC3 cells.

H. UMAP representation of TC-NER signature (top), expression of POLR gene family of TC-NER signature (bottom).

I. Top expressed gene members in POLR gene family (top). UMAP representation of POLR2L gene expression (bottom).

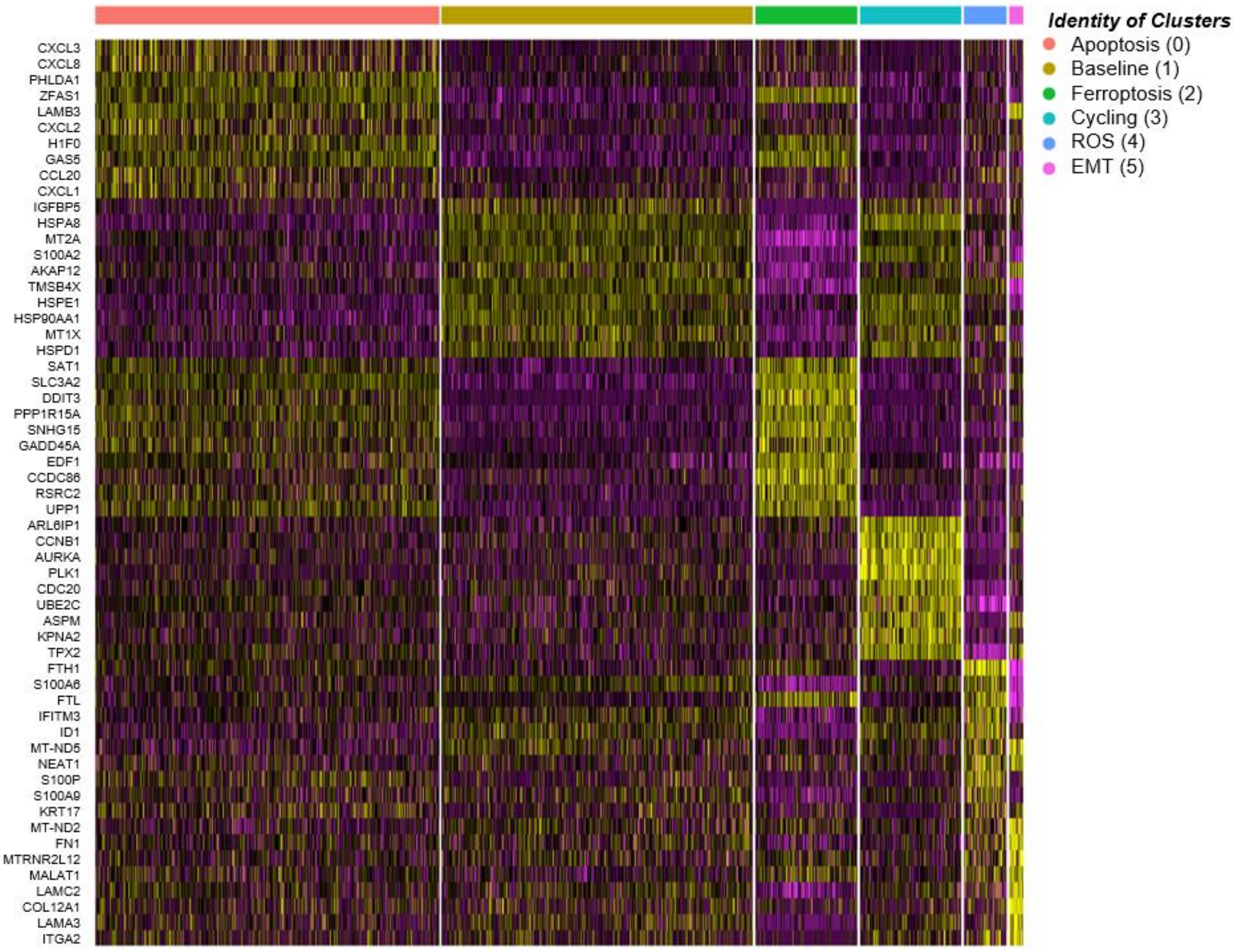

Supplemental Figure 19 | Heatmap of top 10 differentially expressed genes in each cluster when compared with rest of the clusters. Dark yellow- highest expression, dark magenta- lowest expression.

Supplementary Tables

Cohort	Sequencing type	Number of samples
**TCGA-STAD**	WGS	32
**ICGC-GACA**	WGS	36
**ICGC-ESAD**	WGS	97
**TCGA-COAD**	WGS	46
**TCGA-READ**	WGS	16
**TCGA-READ**	WES	172
**TCGA-COAD**	WES	461
**TCGA-STAD**	WES	441
**TCGA-ESCA**	WES	185

Supplemental Table 1. Number of samples analyzed from each cohort. The actual number of samples presented in the results may differ due to the applied filters.

Patient ID	Gene	Chr	POS	REF	ALT	Origin	Function	Genotype	Cohort
**TCGA-BR-4191**	BRCA2	13	32930687	C	T	Germline	Stop gain	Heterozygote mutant with LOH	TCGA-STAD
**TCGA-BR-4267**	BRCA1	17	41209080	-	G	Germline	Frameshift insertion	Heterozygote mutant with LOH	TCGA-STAD
**TCGA-D7-A4YT**	BRCA2	13	32913285	TCAA	-	Germline	Frameshift deletion	Compound heterozygote mutant	TCGA-STAD
**TCGA-D7-A4YT**	BRCA2	13	32339699	-	A	Somatic	Frameshift insertion	Compound heterozygote mutant	TCGA-STAD
**TCGA-IN-A6RL**	BRCA2	13	32363286	C	A	Somatic	Stop gain	Heterozygote mutant with LOH	TCGA-STAD
**TCGA-VQ-A91S**	BRCA1	17	41258474	T	C	germline	SNP	Heterozygote mutant with LOH	TCGA-STAD
**TCGA-L5-A8NS**	BRCA2	13	32914767	TT	-	Germline	Frameshift deletion	Heterozygote mutant with LOH	TCGA-ESCA
**TCGA-AA-3875**	BRCA2	13	32954181	G	A	Germline	SNP	Heterozygote mutant with LOH	TCGA-COAD
**TCGA-CG-4437**	PALB2	16	23649207	ACAA	-	Germline	Frameshift deletion	Heterozygote mutant with LOH	TCGA-STAD
**TCGA-EQ-A4SO**	PALB2	16	23632683	C	T	Germline	Stop gain	Heterozygote mutant with LOH	TCGA-STAD
**TCGA-HU-A4H5**	PALB2	16	23640545	G	A	Germline	Stop gain	Heterozygote mutant with LOH	TCGA-STAD
**TCGA-VQ-AA64**	PALB2	16	23647109	-	A	Germline	Frameshift insertion	Compound heterozygote mutant	TCGA-STAD
**TCGA-VQ-AA64**	PALB2	16	23629212	C	A	Somatic	Stop gain	Compound heterozygote mutant	TCGA-STAD

Supplemental Table 2. The identified BRCA1/2 and PALB2 deficient cases for the TCGA-STAD WES, TCGA-ESCA WES, TCGA COAD WES and TCGA READ WES cohorts.

**Demographics of DFCI cohort**

	N=87 (%)
**Age (median)**	62 years (range: 22-81)
**Gender**	
Female	17 (20)
Male	70 (80)
**Race**	
Asian	3 (3)
Black	1 (1)
Other	2 (2)
White	81 (93)
**Chemotherapy**	
FOLFOX/CapeOX	56 (64)
FOLFOX + trastuzumab	26 (30)
FOLFOX + nivolumab	1 (1)
FOLFOX + VEGF/other	4 (5)
**Site of origin**	
Esophagus	78 (90)
Stomach	9 (10)
**Stage of disease**	
Stage IV	87 (100)
FOLFOX: Folinic acid, 5-fluorouracil, oxaliplatin; CapeOx: capecitabine, oxaliplatin; VEGF: vascular endothelial growth factor	

Supplemental Table 3. Demographics and clinical information for DFCI patients treated with platinum chemotherapy.
